# Cross-tissue patterns of DNA hypomethylation reveal genetically distinct histories of cell development

**DOI:** 10.1186/s12864-023-09622-9

**Published:** 2023-10-19

**Authors:** Timothy J. Scott, Tyler J. Hansen, Evonne McArthur, Emily Hodges

**Affiliations:** 1 Vanderbilt Genetics Institute, Vanderbilt University School of Medicine, Nashville, TN 37232 USA; 2 Department of Biochemistry, Vanderbilt University School of Medicine, Nashville, TN 37232 USA; 3 Present Address: Section of Genetic Medicine, Department of Medicine, University of Chicago, Chicago, IL, 60637 USA; 4 Present Address: Department of Medicine, University of Washington, Seattle, WA, 98195 USA

**Keywords:** DNA hypomethylation, Enhancer methylation, Epigenetics, Partitioned heritability, Cell history

## Abstract

**Background:**

Establishment of DNA methylation (DNAme) patterns is essential for balanced multi-lineage cellular differentiation, but exactly how these patterns drive cellular phenotypes is unclear. While > 80% of CpG sites are stably methylated, tens of thousands of discrete CpG loci form hypomethylated regions (HMRs). Because they lack DNAme, HMRs are considered transcriptionally permissive, but not all HMRs actively regulate genes. Unlike promoter HMRs, a subset of non-coding HMRs is cell type-specific and enriched for tissue-specific gene regulatory functions. Our data further argues not only that HMR establishment is an important step in enforcing cell identity, but also that cross-cell type and spatial HMR patterns are functionally informative of gene regulation.

**Results:**

To understand the significance of non-coding HMRs, we systematically dissected HMR patterns across diverse human cell types and developmental timepoints, including embryonic, fetal, and adult tissues. Unsupervised clustering of 126,104 distinct HMRs revealed that levels of HMR specificity reflects a developmental hierarchy supported by enrichment of stage-specific transcription factors and gene ontologies. Using a pseudo-time course of development from embryonic stem cells to adult stem and mature hematopoietic cells, we find that most HMRs observed in differentiated cells (~ 60%) are established at early developmental stages and accumulate as development progresses. HMRs that arise during differentiation frequently (~ 35%) establish near existing HMRs (≤ 6 kb away), leading to the formation of HMR clusters associated with stronger enhancer activity. Using SNP-based partitioned heritability from GWAS summary statistics across diverse traits and clinical lab values, we discovered that genetic contribution to trait heritability is enriched within HMRs. Moreover, the contribution of heritability to cell-relevant traits increases with both increasing HMR specificity and HMR clustering, supporting the role of distinct HMR subsets in regulating normal cell function.

**Conclusions:**

Our results demonstrate that the entire HMR repertoire within a cell-type, rather than just the cell type-specific HMRs, stores information that is key to understanding and predicting cellular phenotypes. Ultimately, these data provide novel insights into how DNA hypo-methylation provides genetically distinct historical records of a cell’s journey through development, highlighting HMRs as functionally distinct from other epigenomic annotations.

**Supplementary Information:**

The online version contains supplementary material available at 10.1186/s12864-023-09622-9.

## Background

Among the twenty-eight million CpG dinucleotides in the human genome, the majority (80–85%) of cytosines undergo constant DNA methylation (DNAme) in most cellular contexts [1–5]. However, a subset of sites forms discrete regions containing stretches of CpGs that are not covalently modified by methylation and are thus considered “hypomethylated”. The majority of these hypomethylated regions (HMRs) are non-coding and coincide with putative gene regulatory elements including promoters and enhancers [5–9].

DNAme has long been tied to transcriptional control; however, apart from a very small subset of developmentally regulated genes, promoters are stably hypomethylated and largely invariant across cell types, regardless of gene transcriptional status [10–13]. Thus, promoter HMRs poorly predict transcriptional programs that ultimately determine cellular phenotypes. By contrast, enhancer HMRs vary considerably between cell types, which results from their context-dependent demethylation [14–22]. While enhancer HMRs are more predictive of nearby gene activity than promoter HMRs [8], how these HMRs are established or maintained and their relationship to cell identity is not well understood.

We previously showed that non-coding HMRs represent an exclusive subset of chromatin accessible sites [8]. More recently, we showed that, while HMRs correlate with chromatin accessibility and other indicators of permissive chromatin, the temporal dynamics of HMR formation is distinct from chromatin remodeling changes [20, 23]. Importantly, HMRs can persist long after chromatin remodeling changes during cell fate transitions in terminally differentiating hematopoietic cells [20, 23]. Similarly, in the mammary gland, gene regulatory changes during the first pregnancy result in demethylation of pregnancy-responsive gene enhancers. The maintenance of these enhancer HMRs is long-lasting, even after pregnancy signals dissipate [24]. These studies indicate that HMRs capture both active and previously active gene regulatory elements in a manner not reflected by other common enhancer-associated chromatin states.

Despite these observations, very few studies have considered the combinatorial and temporal significance of HMR patterns in a genome-wide manner across developmentally diverse datasets. For example, “super-enhancers”, a class of enhancers that are defined by high levels of histone H3 lysine 27 acetylation (H3K27ac) and Mediator binding, are often comprised of multiple enhancers units [25, 26]. Both selective and persistent hypomethylation of individual enhancer units within super-enhancers have been observed in mouse embryonic stem cells (ESCs) during exit from naïve pluripotency [27, 28]. These combinations of HMR patterns suggest that coordinated hypomethylation of enhancers through cell fate transitions serves to uphold specific cellular states. Altogether, this argues that HMRs are established and maintained as a memory of gene regulatory activity; thus, consideration of how HMRs are shared within and between cell types may inform critical epigenetic patterns that secure cellular phenotypes. However, this hypothesis and its link to phenotypic outcomes remains to be tested across diverse tissues and developmental timepoints in a genome-wide manner.

Here, we performed a comparative analysis of whole-genome methylation data from diverse tissues representing distinct organ systems and developmental timepoints. Unlike previous studies that emphasize pairwise differential methylation or locus-specific changes during limited differentiation time courses, we comprehensively characterize HMR relationships both within and between cell types to understand the functional significance of combinatorial HMR patterns. By analyzing methylomes across diverse cell types, both distant and related, we show that hierarchical conservation of HMRs across tissues can identify enhancer HMRs established in developmentally distinct contexts. We further demonstrate that HMRs established at distinct timepoints partition the genome in a way that is highly predictive of complex trait heritability, which highlights the significance of these HMR patterns to the underlying genome sequence. Ultimately, these data provide novel insights into how DNA hypo-methylation informs genome function by providing a map that traces the developmental histories underlying cellular states.

## Results

### Shared HMR patterns among diverse cell types reveal common functional and developmental histories

**Fig. 1:**
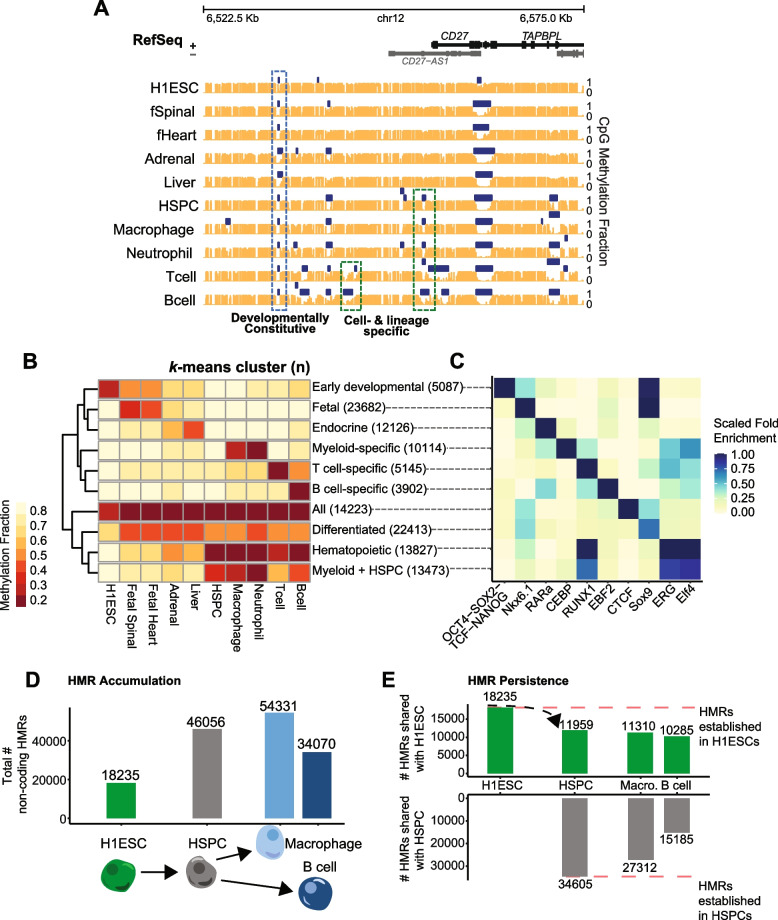
Levels of HMR specificity recapitulate developmental relationships through accumulation and maintenance. **A** Multiple alignment of WGBS methylation and HMR tracks across 10 cell types: H1 ESC, fetal spinal cord, fetal heart, adrenal gland, liver, hematopoietic stem and progenitor cells, neutrophil, macrophage, B cell, and T cell. Methylation tracks are represented by orange vertical bars showing methylation value per CpG site. Methylation fraction is calculated as the fraction of reads containing a methyl-C over the total number reads covering a CpG site. HMR calls are shown by dark blue horizontal bars. Developmentally constitutive, lineage-specific, and cell specific HMRs are highlighted by blue and green dotted bars, respectively. The *plotgardener* R package was used to generate the genome browser snapshot [29]. **B** Heatmap of average methylation per HMR across cell types. Non-coding HMRs were *k*-means clustered based on their average CpG methylation values across 10 cell types represented in (**A**). A *k*-means of 10, assessed by the elbow method, was used to cluster HMRs into groups that are consistent with the biological relationships of their cell types. Groups are manually labeled to reflect their biological relationships. **C** The transcription factor (TF) motif enrichment of each *k*-means group reflects biological relationships captured in (**B**). Representative TFs were selected from the top significant hits ranked by natural log adjusted *p*-value for each *k*-means group. The top ranked TFs are shown unless the top TF(s) for that group were redundant; the second top ranked TF is shown for the group, “Myeloid + HSPC,” and the third ranked TF is shown for the group, “Differentiated.” Fold enrichment values are normalized from 0 to 1 across TFs. The background comparison file comprises HMRs across all represented cell types. **D** Bar graph of the total number HMRs for each cell type, arranged by developmental progression. **E** Bar graph measuring the presence of HMRs established in either H1 ESCs (*top; green)* or HSPCs (*bottom; grey*) in developmentally progressive cell types. The software *Bedtools intersect* was used to determine overlap between cell type HMR datasets using default settings [30]. Overlap was defined as a 1 bp minimum

Studies aiming to understand the relationship between DNA methylation patterns and phenotypic outcomes have focused largely on individual differentially methylated regions without consideration of combinatorial changes that drive phenotypes. To understand the functional significance of complex HMR patterns, we determined the correspondence of HMRs across diverse human cell types and developmental timepoints. We hypothesized that shared HMR patterns among diverse cell types could reveal common functional and developmental histories. To illustrate this idea, genome browser tracks of methylation data are displayed for datasets representing diverse lineages and developmental timepoints at a B cell enhancer cluster upstream of the *CD27* gene (Fig. 1A). This locus contains a group of HMRs with varying levels of HMR specificity that are surrounded by genes involved in lymphoid development and signaling including *CD27*, *LTBR*, and *TAPBPL*. A comparison of HMRs reveals different levels of both cell-type and lineage specificity, including HMRs conserved in all samples (developmentally constitutive); HMRs shared exclusively among lineage-related samples (e.g., hematopoietic cells); and HMRs present only in B cells. The lymphocyte-specific expression of *CD27* highlights a potentially important role for the combination of shared and cell specific HMRs observed at this locus.

To investigate the extent to which these HMR patterns can be observed globally, we determined a set of high-confidence HMRs using publicly available whole genome bisulfite sequencing (WGBS) data from ten different cell types and tissues, including embryonic stem cells (H1 ESCs), hematopoietic stem & progenitor cells (HSPCs), fetal heart, fetal spinal cord, liver, adrenal gland, macrophages, neutrophils, T cells, and B cells (see Methods). As the resolution of HMR specificity is contingent on the quantity and interrelatedness of cell types included in the analysis, we maximized comparative potential by including datasets representing a diversity of organ systems and developmental stages.

HMRs were determined for each dataset using MethPipe [31, 32], which employs a computational model originally described in Molaro et al. 2011 [18] to detect adjacent clustering of unmethylated CpG sites in the genome. Specifically, a 2-state hidden Markov model (HMM) with Beta-Binomial emission distributions allowed high and low methylation states to be trained separately on each individual WGBS dataset [31, 32]. This modeling approach is robust to sequence coverage differences both within and between WGBS datasets. This is important given that sequence coverage is not uniformly distributed across the genome. Therefore, we required a minimum mean sequence read coverage of 10x at symmetric CpG sites for any HMR dataset to be included in our analysis. Of the ten datasets included, eight achieve CpG read coverage > 25x, while the B cell and neutrophil datasets reach nearly 12x (Table S1) [31]. This resulted in a total set of 126,104 unique non-coding HMRs with an average length of ~ 866 bp (Fig. S1). Those HMRs spanning transcriptional start sites (TSS; -2000/ + 1000 bp) and exons were excluded from the analysis in order to focus on non-coding HMRs harboring putative enhancers (Table S2). By excluding the substantial number of constitutive HMRs overlapping gene promoters, we achieve better resolution to detect non-promoter HMR patterns that contribute to cellular states.

Hierarchical clustering applied to these datasets was sufficient to recapitulate both related and distant cell type relationships, demonstrating the quality and specificity of HMR calls (Fig. S2). Next, we utilized *k*-means clustering to group HMR methylation levels across the 10 different cell types and tissues in an unsupervised manner. We used the elbow method to determine an optimal number of *k*-means clusters (*n* = 10, Fig. S3).

The resultant heatmap revealed groups of HMRs highly stratified by both group function and developmental stage (Fig. 1B). We manually classified each *k*-means group according to cell types displaying average HMR methylation ≤ 50% for each group. For example, in the “Hematopoietic” HMR group, blood cells uniquely display low methylation levels, whereas the “Early Developmental” HMR group is dominated by H1 ESCs. Likewise, a group of HMRs is specific to the “Fetal” developmental state compared to stem and adult cells. Using this analysis, we achieve remarkable resolution to distinguish HMRs that are unique between highly related cell types such as macrophage and neutrophil cells; further, we identify a more specific group of exclusive T and B cell HMRs.

Since transcription factors (TFs) govern the functional progression and specialization of cell types, we performed TF motif enrichment analysis to understand the gene regulatory significance of each *k*-means group. Top motifs stratify strongly by *k*-means group (Fig. 1C). Furthermore, representative TFs from top results show *k*-means group-specific enrichment of canonical TFs indicative of their respective cell types. For example, the ubiquitous CTCF is enriched in the *All* group [33]; pluripotency factors OCT4-Sox2-Nanog are primarily in the *Early Developmental k*-means group [34]; CEBP, a factor important for myeloid development, is enriched exclusively in macrophages and neutrophils (myeloid cells) [35]; and early B cell factor EBF2 is in the highly specific *B cell* group [36]. Similarly, the retinoic acid receptor alpha (RARα) motif is highly enriched exclusively in the liver/adrenal-specific *Endocrine* group. The enrichment of cell specific transcription factors in cell specific HMRs confirmed expectations of our HMR group annotation strategy and highlights the ability to observe shared HMR patterns that reflect not only subsets of cell types but also developmental periods.

Given the specificity of the TF enrichment analysis supporting cell- and lineage-specific functions, we considered whether associated genes displayed similar biological specificity. We used GREAT ontology enrichment analysis to analyze sets of genes neighboring HMR groups defined by *k*-means clusters shown in Fig. 1B [37]. We show enrichment of distinct biological processes representative of the cell type and developmental stage associated with HMR groups (Fig. S4). Interestingly, the least differentiated HMR group of *Early development* enriched for early morphogenic specification ontologies, while the intermediate HMR group defined by sharing between the blood cell types and stem and progenitor cells enriches for blood-related signaling ontologies. Additionally, the myeloid-specific enrichments show myeloid lineage specificity, whereas B cell-specific ontologies are enriched in the *B cell* group; this highlights the ability of HMRs to distinguish not only disparate lineages and developmental stages, but also highly related cell types. Together these data show that HMRs alone can recapitulate functional relationships between cell types. Furthermore, by comparing HMRs within and across lineages, we discovered that levels of HMR specificity can reflect deep developmental roots of gene regulation, capturing time point-specific branchpoints of development (Fig. 1 and S2). For example, the hematopoietic *k*-means cluster contains a group of HMRs that are shared between stem and progenitor cells as well as derived cell types (B cell, T cell, neutrophil, and macrophage), but not others. This data suggests that HMRs established at specific, early developmental timepoints are maintained in subsequent cellular states. We explore this in further detail below.

### HMRs accumulate and persist through subsequent developmental transitions

Terminally differentiated cells exhibit between ~ 2–3.5 times the number of non-coding HMRs compared to embryonic stem cells (Fig. 1D). While a minor subset of H1 ESC HMRs are cell type-specific, most H1 ESC HMRs are highly shared across the cell types analyzed (Fig. 1B, 14,223 merged HMRs that are shared among “All” cell types; of 18,235 H1 ESC non-coding HMRs, 2,616 are cell specific while 15,619 are shared with at least one other cell type, a 5.97-fold difference). Our comparative analysis further reveals specific HMR groups defined by developmental stage (fetal vs. adult, differentiated vs. undifferentiated), lineage, and cell type (Fig. 1B). These data suggest a model whereby H1 ESCs supply a base HMR set to which additional HMRs are added at distinct lineage commitments through cell development. This is important because it suggests that a developmental hierarchy exists among HMRs and that HMRs accumulate as cells differentiate.

To determine whether progressive HMR establishment can be traced in developmentally derived cell types, we used pluripotent H1 ESCs, multipotent HSPCs, and terminally differentiated myeloid (macrophages) and lymphoid (B cells) lineage cells to construct a pseudo-time course (Fig. 1D). In general, we observe that non-coding HMRs increase in number with increasing cell maturity. An increase of total HMRs could be explained by 1) a simple accumulation of additional HMRs, or 2) a net increase with high turnover of HMRs. To differentiate between these two modes of HMR expansion, we measured HMR overlap between either embryonic stem cells or hematopoietic stem cells and mature hematopoietic cell types. Of 18,235 HMRs observed in H1 ESCs, 11,959 (65.58%) were represented by HMRs in the total multipotent HSPC dataset. Of these 11,959 HMRs that were observed in both H1 ESCs and HSPCs, 11,310 (62.02%) and 10,285 (56.40%) were represented by HMRs in the macrophage and B cell datasets, respectively (Fig. 1E). Next, of 34,605 HMRs established in HSPCs but absent in H1 ESCs, 27,312 (78.93%) and 15,185 (44.23%) were represented by HMRs in the macrophage and B cell datasets, respectively (Fig. 1E).

These data show that a majority of the HMRs observed in differentiated cells (~ 60%) are established at early developmental stages and suggest a pattern of HMR accumulation in relation to developmental progression. In addition to acquiring new HMRs, macrophages retain a majority of HMRs established in HSPCs, whereas B cells retain half as many HSPC-derived HMRs and fewer total HMRs compared to macrophages. This observation is consistent with previous studies demonstrating that lymphoid commitment and myeloid restriction requires re-methylation of specific early hematopoietic regulatory elements in parallel to demethylation of lymphoid-specific elements [38–40]. Failure to remethylate these regions can result in a lineage priming imbalance favoring myeloid differentiation; thus, fewer HMRs are retained from HSPCs in B cells compared to macrophages. Despite this B cell remethylation of a subset of HSPC HMRs, we observe a general increase in HMRs across the hematopoietic lineage that supports a model where new HMRs are progressively established through successive developmental stages and persist through later stages of cell differentiation.

### HMRs are non-randomly established into spatially organized clusters

**Fig. 2:**
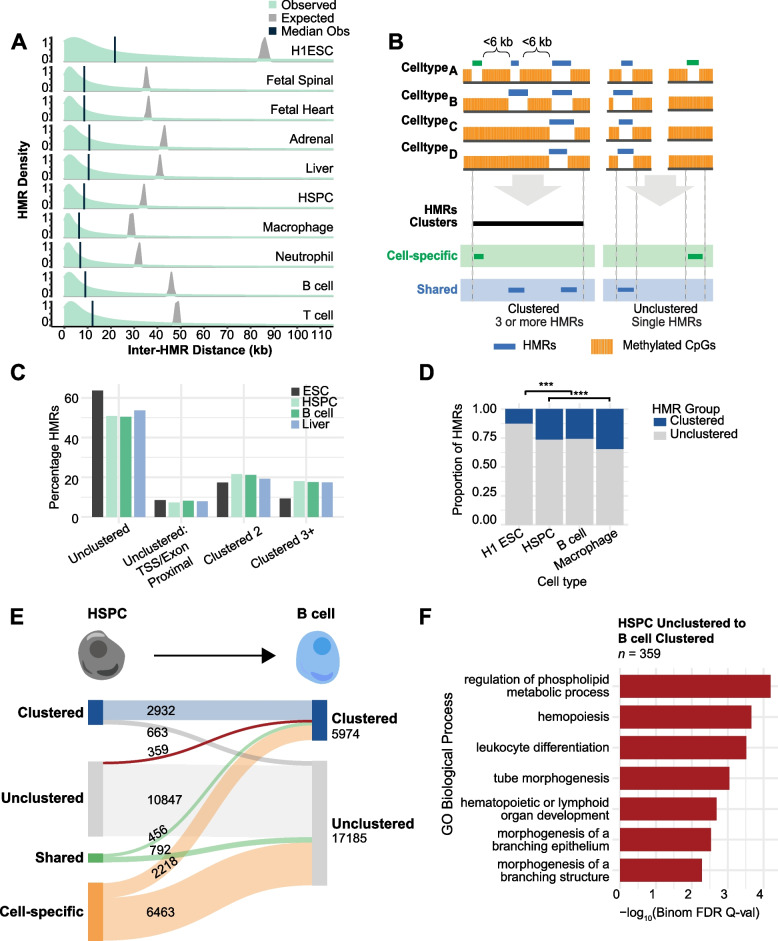
HMRs cluster more than expected. **A** Distribution plots of inter-HMR distances by cell type. The green distributions represent observed values from HMR datasets per cell type. Vertical navy bars show median values. Grey distributions show expected values by random shuffling across the non-coding genome. For each cell type, the expected and observed distributions were determined to be significantly different by the Wilcoxon rank sum test. All *p*-values were reported as zero (*p* < 2e-16) with a range of Χ^2^ values from 1.4337 × 10^8^–4.4508 × 10^8^. **B** Diagram of HMR clustering and cell specificity workflow. HMRs are annotated for clustering behavior and/or cell specificity. Non-coding HMR datasets are defined by HMRs that do not overlap RefSeq protein-coding TSSs (TSS -2000/ + 1000) and exons. Clustering refers to groups of HMRs in a cell type that are located a maximum of 6 kb end-to-end from the next HMR, linking 3 or more HMRs; clusters cannot cross TSSs or exons. Unclustered HMRs are defined as non-coding HMRs that are not within 6 kb of any other non-coding or TSS/exon-overlapping HMR. Cell specificity is also defined, with any base pair overlap between HMRs constituting overlap. **C** Bar graph of HMR clustering annotations discussed in (**B**) and Fig. S6 as percentages of total HMRs by cell type. Selected cell types represent members of the hematopoietic and hepatic lineages. Colors reflect cell types representing different developmental stages and lineages. **D** Bar graph of proportion of cell type HMRs that are clustered HMRs (3 + HMRs) vs unclustered. Total values are calculated as [#unclustered + #clustered]. **E** Sankey diagram showing the flow of B cell HMRs. B cell HMRs are divided on the right of the panel into clustering groups. The left shows HSPC HMRs that overlap B cell HMRs, and are hierarchically categorized as *clustered HSPC* *HMR*, *unclustered HSPC HMR*, *shared*, or *cell specific*. To define cell specificity, B cell HMRs were compared to datasets from adrenal gland, H1 ESC, HSPC, fetal spinal, fetal heart, liver, macrophage, neutrophil, and T cell*.* **F** The bar graph shows the top biological process gene ontology results for the Sankey group of HMRs that progress from *HSPC unclustered* to *B cell clustered* (indicated in red). Results from GREAT Gene Ontology using default background and gene assignment settings are represented by bars showing binomial *q*-value [37]

Locus-specific analysis of individual WGBS datasets indicates that multiple distinct HMRs are frequently located near one another, rather than being randomly distributed across linear genomic space (Fig. 1A). Moreover, these HMR groups appear to be spatially organized with HMRs that are present in varying degrees of cell types and tissues, from developmentally constitutive to B cell specific. The example locus shown in Fig. 1A depicts a group of adjacent HMRs near the *CD27* gene. *CD27* and several other genes surrounding the locus play a key role in B cell function [41–43]. To quantify this HMR grouping phenomenon genome-wide, we calculated observed and expected distributions of inter-HMR distances utilizing the cell types represented in Fig. 1. Expected distributions were simulated by random shuffling (*n* = 10,000) HMRs across the genome for each dataset, excluding a blacklist of protein coding RefSeq TSSs (-2000/ + 1000 bp) and exons. HMR distances are significantly closer to each other than expected by random chance (Fig. 2A, Wilcoxon rank sum, *p*-value < 2.2e^−16^). Interestingly, differentiated cell types consistently show lower expected and observed distances (~ 40–50 kb and ~ 12 kb, respectively) compared to those of H1 ESCs, which feature the largest inter-HMR distances; this is consistent with having fewer HMRs overall, supporting its role as a basal HMR set.

These data suggest that clustered HMRs play a distinct regulatory role compared to their unclustered counterparts. To characterize the features that distinguish “clustered” and “unclustered” HMRs we first determined a set of heuristic criteria to define clusters (Fig. 2B). We plotted per-cell type distributions for non-coding inter-HMR distances and measured distance quantiles. From this, we analyzed "end-to-end" cluster lengths based on three maximum linking values: the ≤ 12.5 kb stitching distance commonly used in ChIP-seq-based super-enhancer studies [25, 26, 44–46]; the approximate mean inter-HMR distance of 11 kb and ≤ 6 kb which represents the median inter-HMR distance after filtering for values under 50 kb (Table S3). Previous studies that have characterized clustered super-enhancers have used a common linking distance threshold of 12.5 kb. This distance was reportedly selected for its ability to qualitatively link high signal regions together while avoiding inclusion of lower signal peaks. Thus, the super-enhancer definition is reliant upon signal intensity and distribution. However, HMRs are defined by a bimodal methylation signal distribution, and such a distance applied to methylation data results in extraneously long stitched regions, the biological function of which is difficult to assign; some exceed 1 Mb, which can result from HMRs spread across gene deserts, or large topological domains with low methylation levels or CpG frequency. By comparison, a linking distance of 6 kb results in stitched regions with an overall mean length of ~ 10 kb, which is consistent with other clustered enhancer annotations such as stretch and super-enhancers (Table S4, Fig. S5) [26, 47]. Using a linking distance of 6 kb, we determined the fraction of HMRs that are clustered or unclustered for a subset of cell types, including H1 ESC, HSPC, B cell and Liver (Fig. 2C). To avoid confounding contributions of promoter characteristics to our analysis, clusters were not allowed to cross TSSs or exons. At a 6 kb threshold, non-TSS/exon HMR groupings that exist as pairs or as clusters of 3 or more constitute ~ 35% of all HMRs. For the rest of this paper, “clusters” refer to clusters with 3 or more HMRs (see annotation strategy in Fig. S6).

As demonstrated in Fig. 1A a typical cluster consists of multiple HMRs with different levels of cell type specificity between them—broadly shared (developmentally constitutive), lineage-shared or cell-specific. This means that a cluster identified in one cell type may not exist across all cell types. As the formation of clusters is contingent on the addition of new HMRs near existing HMRs, most HMR clusters (~ 35–40%) contain at least one lineage- and/or cell type-specific HMR. Given that HMRs accumulate over developmental timelines, this observation raises the possibility that, as cells differentiate, HMRs are preferentially added to clusters in a lineage-specific manner. Indeed, we observe a positive correlation between clustering and developmental state. Clustering percentage increases as development progresses (Fig. 2D, *H1 ESC to HSPC* & *HSPC to Macrophage*: *p* < 2.2 × 10^–16^), and this is accompanied by a relative decrease in unclustered HMRs. Tracking these HMRs temporally for each pseudo-timepoint reveals that a substantial fraction of early-established HMRs is joined by additional HMRs in subsequent developmental states. The establishment of new HMRs near existing HMRs can lead to clustering, where an HMR may be classified as unclustered at an early timepoint but become clustered in a differentiated cell type (Fig. 2E). These growing clusters of HMRs are often in proximity to lineage-specific genes, as suggested by gene ontology analysis (Fig. 2F). Altogether, these data show that HMRs can be broadly distinguished by 1) the number of cell types that share them—a corollary of temporal establishment or developmental time—and 2) their clustering behavior, which may reflect a collective and unique developmental function that distinguishes clustered HMRs from other types of genomic regions.

### Clustered HMRs are functionally distinct from unclustered HMRs

Sequencing approaches have enabled the discovery of many spatially clustered regulatory elements genome-wide using chromatin accessibility [47], histone modifications [48–53], and transcription factor binding [25, 26]. More recently, clustering of enhancers has been commonly associated with concepts such as super-enhancers (SEs) [26], stretch enhancers [47], shadow enhancers [54, 55] and locus control regions (LCR) [56], which are thought to provide regulatory additivity, synergy, and redundancy to their target genes in a tissue-specific manner.

**Fig. 3:**
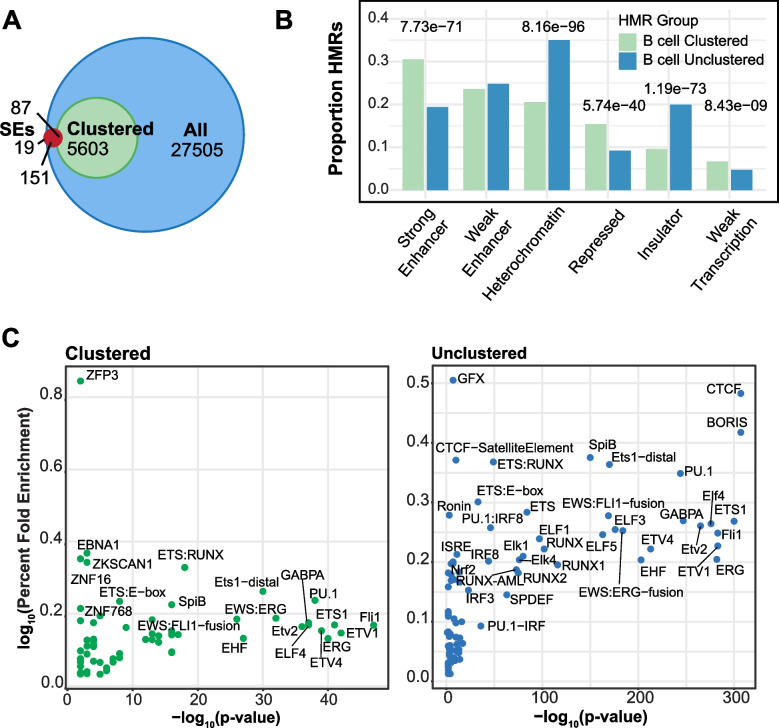
Clustered HMRs show distinct enhancer-associated characteristics compared to unclustered HMRs. **A** Venn diagram showing partially overlapping sets between three region datasets: All B cell HMRs (blue circle); clustered B cell HMRs (green circle; subset of All); and GM12878 super-enhancers (red circle) [26]. GM12878 is a tier 1 ENCODE lymphoblastoid cell line derived from EBV immortalized B cells. **B** Bar graph of HMR overlap with selected ChromHMM annotations: *strong enhancer*, *weak enhancer*, *heterochromatin*, *repressed*, *insulator*, *weak transcription*. The height of the bars represents the fraction of clustered and unclustered HMRs that overlap each annotation. Z-test of proportion *p*-values are shown, comparing HMR group proportion values for each ChromHMM annotation. **C** TF motif enrichment in clustered (left; green) and unclustered (right; blue) HMRs. Results are plotted as -log_10_*p*-value by fold enrichment, measured as percentage of target regions containing motif divided by the percentage of background regions. Background represents all clustered and unclustered HMRs

Comparison of clustered B cell HMRs with histone H3K27ac-defined B cell super-enhancers shows that, while the majority of super-enhancers coincide with HMRs (both clustered and unclustered), only 1.5% of clustered HMRs overlap super-enhancers (Fig. 3A) [26]. This discrepancy may be explained by the observation that only a fraction of SEs exists as clusters in linear genomic space. Indeed, 15% of SEs from Whyte et al. are singletons and only 196 of 1,355 stitched murine ESC enhancers are SEs [25, 57]. Thus, super-enhancers do not exclusively consist of clustered enhancers, and ChIP-seq defined enhancer clusters are not exclusively SEs. Clustered HMRs are more frequent than SEs, and their existence raises the question of whether they represent distinct functional characteristics compared to their unclustered HMR counterparts.

To address this question, we used ChromHMM annotations to functionally categorize HMRs based on clustering behavior in B cells (Fig. 3B) [48, 49]. Notably, clustered HMRs are enriched for “strong enhancers” (*X*^2^ = 316.66, *p* = 7.725 × 10^–71^) while unclustered HMRs show higher enrichment of “heterochromatin” (*X*^2^ = 432.37, *p* = 8.159 × 10^–96^) and “insulators” (*X*^2^ = 329.57, *p* = 1.191 × 10^–73^). This suggests clustered HMRs are enriched for active regulatory regions while unclustered HMRs tag elements involved in three-dimensional chromatin structure. This result is corroborated by the strong enrichment of the CTCF motif in unclustered HMRs, while both clustered and unclustered B cell HMRs show comparable enrichment of lymphoid-relevant transcription factors, including PU.1, SpiB, and ETS family members (Fig. 3C).

**Fig. 4:**
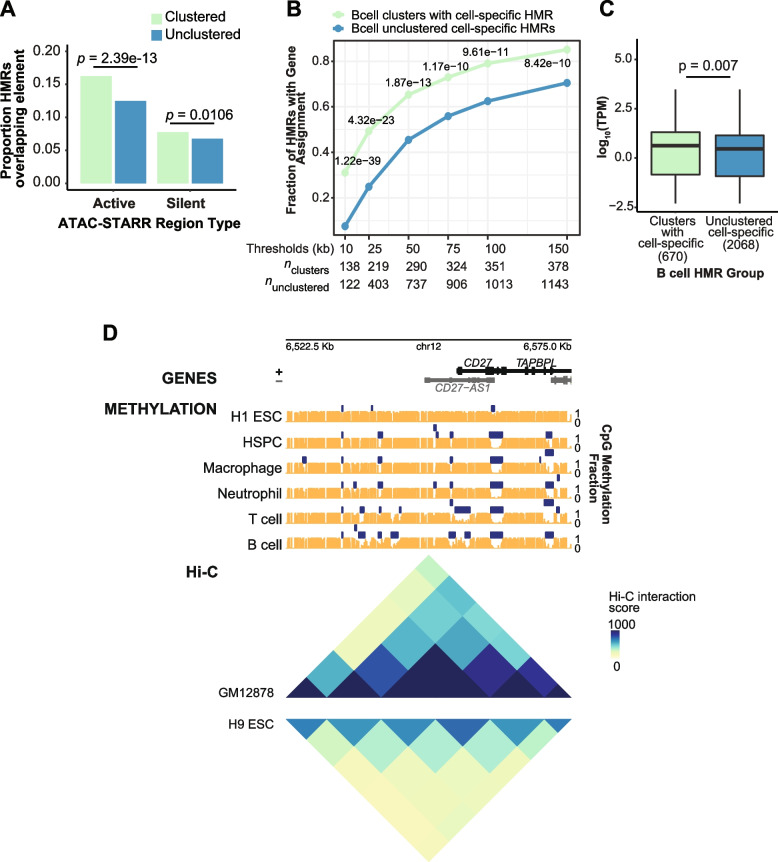
Clustered HMRs are enriched for active regulatory elements compared to unclustered HMRs. **A** Boxplot of ATAC-STARR-seq regulatory element overlap by clustered and unclustered HMRs. Overlap is measured at the unit of HMRs, and values depict fraction of total HMRs that contain a regulatory element. A Wilcoxon rank sum test was used to determine statistical significance. **B** Point and line graph of the proportion of HMRs near an expressed gene at different TSS distances. HMRs are grouped by HMR clusters that contain a cell specific HMR and unclustered cell specific HMRs. Denominators for the HMR clusters and unclustered HMR groups are 444 and 1621, respectively. Counts below the graph represent the cumulative amount of genes below each threshold per HMR group. *p*-values are derived from a z-test of proportions to test the fraction of HMRs represented by HMR-single nearest neighbor gene pairs below each threshold distance. **C** Boxplot of TPM values (derived from GM12878 cell line data) of nearest neighbor RefSeq protein-coding genes to clustered and unclustered HMRs. Two nearest neighbor genes (with TPM > 0) per HMR were filtered for TAD boundary crossing. Statistical significance was measured by a Wilcoxon rank sum test in R using the *wilcox.test() function*. **D** Multiple alignment of region around the *CD27* locus showing methylation and HMR tracks across 6 cell types: H1 ESC, hematopoietic stem and progenitor cells, macrophage, neutrophil, B cell, and T cell. Methylation tracks are represented by orange vertical bars showing methylation value per CpG site. HMRs are shown by dark blue horizontal bars. Below the multiple alignment, Hi-C interaction score data is represented by heatmap triangles representing interaction matrices for GM12878s and H9 ESC cells. Values for the Hi-C data are derived from .hic interaction matrix files. The *plotgardener* R package was used to generate the genome browser snapshot [29]

Given the enrichment of strong enhancer annotations in clustered HMRs, we investigated their transcriptional regulatory activity by comparing with our recently published ATAC-STARR-seq data for immortalized B cells (Fig. 4A) [58]. ATAC-STARR-seq is a massively parallel reporter assay that uses Tn5 transposase to selectively clone accessible DNA from native chromatin into a plasmid-based reporter to test accessible chromatin regions for active and silent regulatory activity [58, 59]. Since a majority of B cell HMRs overlap accessible chromatin regions in lymphoblastoid cells (Fig. S7), we measured the proportion of HMRs that contain an activator or silencer (Fig. 4A). Despite being fewer in number, clustered HMRs contain a significantly higher proportion of transcriptional regulators, including both activators and silencers (*p* = 2.39 × 10^–13^ and 0.0106, respectively), than unclustered HMRs.

Based on the finding that clustered HMRs are enriched for both strong enhancer annotations and “activators” defined by ATAC-STARR-seq (Fig. 4A), we hypothesized that clustered HMRs are more likely to be associated with active genes compared to unclustered HMRs. To address this question, we defined pairs of HMRs and their nearest neighbor genes, measuring the proportion of cell-specific clustered and unclustered HMRs that tag the nearest “active” (TPM > 0) gene at different threshold HMR-TSS distances (Fig. 4B). To pair HMRs with their nearest neighbor active gene, we used a gene assignment strategy that identifies the nearest expressed neighboring gene within a topologically associated domain (TAD) containing both the gene and the HMR(s). A recent study showed that a combination of nearest neighbor assignment in conjunction with a minimum expression threshold increased associated-gene prediction accuracy above several gene assignment methods, including the commonly utilized simple nearest neighbor [60]. Using this assignment strategy, we paired HMR groups with lymphoblastoid RNA-seq (GM12878) data from ENCODE as a proxy for B cells [61]. Focusing on B cell specific HMRs, we observed a significantly higher proportion of clustered HMRs near active genes compared to unclustered HMRs at all observed distance thresholds (Fig. 4B; all *p*-values < 8.42e-10). While our unclustered definition omits HMRs that are within 6 kb of TSSs or exons, these observations remain consistent when TSS/Exon proximal HMRs are included (Fig. S8A; all *p*-values < 1.96e-5). Similar results were obtained for the same analysis performed on liver HMRs (Fig. S8B).

We further reasoned that target genes of clustered HMRs display increased transcriptional output compared to those of unclustered HMRs. To define putative HMR target genes, we used a similar approach to the gene assignment strategy discussed above that incorporates both a TAD and expression filter (TPM > 0); due to uncertainty that the nearest gene is a true positive, we considered two nearest neighbors for gene assignment. Using this approach, we observe that genes assigned to clustered B cell HMRs show a significantly higher distribution of transcript levels compared to those near unclustered HMRs (*p* = 0.007; Fig. 4C) [61]. However, when these comparisons are binned by HMR-gene distance, we do not observe significant differences in gene expression across bins, except in the most distal (≥ 100 kb) HMR-gene distance bin (Fig. S8C). We performed the same analysis for liver clustered HMR target genes, finding the pattern is consistent across cell types (Fig. S8D, E). These observations suggest that the functional distinction of clustered HMRs compared to unclustered HMRs is a general phenomenon.

Given the relationship between clustered HMRs and gene activity, we considered whether the appearance of clustered HMRs in differentiated cells accompanies changes in chromatin conformation. We used publicly available Hi-C data to compare long range chromatin contacts around the *CD27* locus between embryonic stem cells and differentiated B cells (Fig. 4D). This locus provides a representative example of a cluster of HMRs that accumulates HMRs with increasing developmental specificity. Here, we observe that the accumulation of immune cell specific HMRs coincides with chromatin conformation changes as indicated by increased frequency of Hi-C interactions (Fig. 4D). As the region accumulates clustered HMRs through cell development, new chromatin contacts are created around the newly established HMRs [62, 63], indicating the functional importance of the spatial proximity of clustered HMRs. Altogether, these results argue that combinatorial HMR establishment and HMR history relates to chromatin conformation changes that accompany cell differentiation (see Discussion).

### Non-coding HMR patterns are highly enriched for genetic variants linked to specific clinical phenotypes

Genome-wide associations studies (GWAS) have demonstrated that a substantial portion of human phenotype-associated single nucleotide polymorphisms (SNPs) is located in functional regulatory elements [64–67]. Integration of GWAS with functional genomic data reveals that disease risk variants also localize primarily within cell type-specific enhancers of disease-relevant tissues [68]. Studies examining the relationships between disease loci and molecular phenotypes such as gene expression, chromatin accessibility or the DNAme status of *cis*-acting enhancers have identified a strong connection between non-coding genetic variants and epigenetic regulation [69–71]. Based on these previous studies, we expected a SNP enrichment among HMR patterns that would associate with various traits. We therefore asked whether specific HMR patterns harbor genetic variants linked to distinct clinical phenotypes, and, in turn, whether these relationships can inform the functional significance of different HMR patterns.

**Fig. 5:**
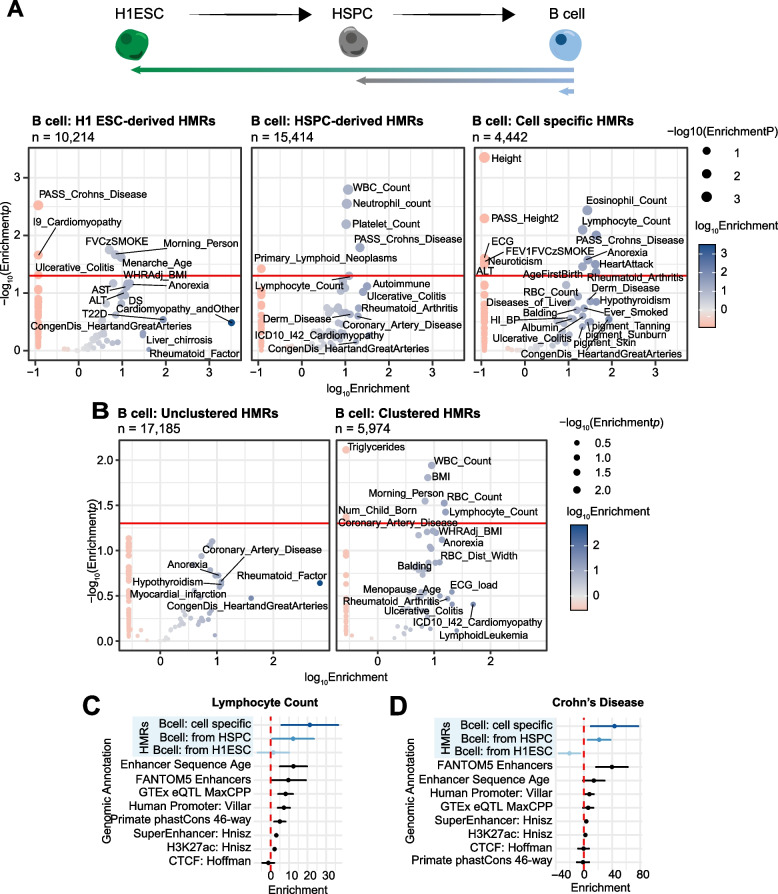
S-LDSC identifies HMR annotation-specific trait enrichments. **A** Volcano-style plots of S-LDSC partitioned heritability results across 79 traits are shown for three *B cell* HMR groups*: H1 ESC-derived, HSPC-derived, and cell specific*. HMRs are ordered by the developmentally distinct cell type in which they were established. Each HMR group was tested for enrichment of genetic heritability with a standard set of 98 base annotations against traits that include both clinical diseases as well as clinical lab values. Negative enrichment values were clipped to the lowest positive enrichment value for each row of plots (A: 0.1174537; B: 0.25754925). The size of each point represents the -log_10_ *p-*value of the enrichment, and the color shows the log_10_ enrichment value. Points with a *p-*value <  = 0.05 or an enrichment > 10 are labeled by their trait name where available. **B** Further partitioned heritability analysis applied to B cell HMRs grouped only by clustering behavior is also represented. **C** Point and line plot of S-LDSC enrichment values by annotation group for “Lymphocyte Count”. These graphs include data from developmentally derived B cell HMRs compared against other enhancer-associated groups, including ancient human enhancer sequence age, FANTOM 5 enhancers, eQTLs, super-enhancers, and the H3K27ac histone mark. Genomic controls were also included, such as phastCons 46-way annotations as well as promoters and CTCF sites. The x-axis represents enrichment values, and the y-axis displays genomic annotations. Points show enrichment estimates and lines display 95% confidence intervals. The red line marks an enrichment score of 0. **D** Point and line plot of S-LDSC enrichment values by annotation group for “Crohn’s Disease”

We reasoned that GWAS SNPs could be leveraged to reveal genetic variants in HMRs of critical importance to normal cell development and function. As in Fig. 1D-E, we defined B cell HMRs that are H1 ESC-derived (developmentally constitutive), HSPC-derived (lineage-shared) or B cell-specific. GWAS SNPs not only reflect trait-associated genetic variation, but also GWAS summary statistics can be used to estimate partitioned genetic heritability of traits assigned to subsets of the genome, based on the assumption that regions with higher quantities of SNPs in high linkage disequilibrium (LD) are more likely to capture a causative variant. We used stratified LD score regression (S-LDSC) to perform partitioned heritability analysis from GWAS summary statistics of 79 traits and clinical lab values representing a range of organ systems ([72] Table S5). We found significant enrichment of trait heritability within lineage- and cell-specific HMRs (Fig. 5A).

More specifically, we found that trait specificity not only stratifies by but also increases with HMR specificity. For example, H1 ESC-derived B cell HMRs are nominally enriched for traits not immediately attributable to B cell function, such as *cardiomyopathy* and *morning person*. This is unsurprising due to the pleiotropy of gene regulation and the shared genetic architecture between many complex traits. However, HSPC-derived HMRs are enriched for genetic heritability of general hematopoietic traits including *white blood cell, platelet,* and *neutrophil counts*. In highly B cell-specific HMRs, we identify a notable enrichment of specific immune-related clinical traits and lab values, several of which achieve significance after multiple testing correction (*p* < Bonferroni, *n* = 79). These observations hold true for S-LDSC analysis in H1 ESC-derived, and cell specific liver HMRs (Fig. S9), reinforcing the notion that cell stage-derived HMRs are indicative of stage-relevant gene regulatory needs.

HMRs stratified solely by clustering behavior also demonstrate heritability enrichment patterns associated with specific lymphoid traits (Fig. 5B). In fact, compared to clustered HMRs, unclustered HMRs show no statistically significant trait enrichment above significance thresholds, suggesting that results in Fig. 5A are powered predominantly by clustered HMRs. Accordingly, these trends were observed in gene-based disease enrichment analyses (disease ontology) applied to the same HMR groups analyzed by S-LDSC (Fig. S10) [73, 74]. For example, the top disease ontology enrichments for H1 ESC-derived HMRs include morphogenic ontologies such as *craniofacial abnormalities*, and the top ontologies for B cell-specific HMRs include multiple lymphoid- and leukemia-related ontologies, reflecting the biological state associated with each HMR group. Together with the partitioned heritability results, these data suggest clustered cell specific HMRs are both near lineage-specific genes and enrich for cell specific trait heritability over that of unclustered HMRs.

To better contextualize the partitioned heritability enrichment results from B cell data, we compared results against other known functional genomic feature annotations. We compared S-LDSC enrichment levels on a per-trait basis for B cell and liver HMR annotations (those from Fig. 5A and Fig. S9) and other functional genomic annotations (Fig. 5C-D, Fig. S11A and B). For both immune-related clinical lab values and disease traits, we observe increasingly stronger enrichment from H1 ESC-derived to HSPC-derived to B cell-specific HMRs. In contrast, both H1 ESC-derived and liver-specific HMRs show positive enrichment for ALT (alanine transaminase*)* compared to B cell HMRs, as expected. This shows that SNP-based trait enrichment is capable of distinguishing HMR patterns from both distant and highly related cell types. Across cell relevant traits, we observe SNP-based heritability enrichment values that surpass those of promoters, expression quantitative loci (eQTLs), and histone marks of open chromatin (H3K27ac) often used to approximate active regulatory regions. Enrichment values associated with cell specific HMRs are comparable to those of FANTOM5 enhancers, supporting the notion that developmentally specific HMRs mark enhancers important for cell identity. Altogether, this analysis highlights the functional significance of different HMR patterns, all of which are enriched for heritability at or above the levels measured for other enhancer definitions. These results further indicate a quantitative relationship between HMR patterns and complex trait heritability. Thus, the stratification of HMRs by “sharedness” between cell types provides important contextual information to predict genome-to-trait relationships.

## Discussion

Here, we use comparative hypomethylation profiling to assess global hypomethylation patterns across cell types. This broader analysis reveals complex patterns of HMR establishment across a developmentally diverse dataset. By examining HMRs in a hematopoietic developmental context, we show that HMRs accumulate at distinct developmental stages and commonly persist through sequential lineage commitments.

**Fig. 6:**
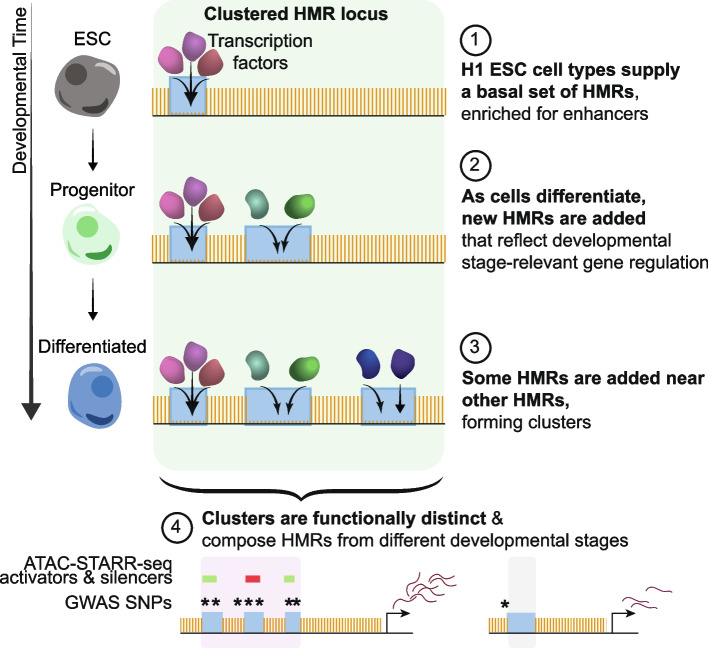
HMRs accumulate in clusters that record histories of cell development. The conceptual model diagram summarizes the observations of HMR accumulation into clusters that feature different levels of methylation specificity

These developmentally hypomethylated regions are associated with distinct, stage-appropriate transcription factors and gene pathways, leading to a model where H1 ESCs, with the fewest HMRs, present a basal set of HMRs to which additional regions are hypomethylated through development (Fig. 6). In fact, about two-thirds of HMRs established in H1 ESCs remain in HMR datasets of differentiated cell types, highlighting their early establishment and continuous hypomethylation across time. Consequently, most (~ 3/4) HMRs in B cells were traced back to either H1 ESCs or HSPCs, indicating that the majority of HMRs are established at early cellular states. This further implies that biological differences between these cell types are driven by the minority population of differentially methylated HMRs. There are some exceptions to this general model, where a small subset of HMRs is “remethylated” between HSPCs and B cells. These regions are likely enhancers of genes involved in myeloid specification, as indicated by their retention in macrophage cells.

Differentially methylated regions (DMRs) can be quantitatively identified and have been commonly used as a unit for studying DNA methylation [75–79]. However, our results demonstrate that consideration of HMRs that are shared among different degrees of developmentally related cell types can be highly informative for understanding the developmental history of the cell. The ability to distinguish unique HMRs between highly related cell types suggests that we can use combinatorial patterns of both shared and unique HMRs to distinguish or even predict cell types, although this remains to be tested.

We further show that new HMRs are preferentially established near existing HMRs, leading to the progressive enrichment of HMR clusters in differentiated hematopoietic cell types; however, it is unclear if these patterns extend to other developmental lineages. Notably, clustered HMRs compose about 1/3 of all HMRs in differentiated cells, compared to less than 1/6 in H1 ESCs, indicating clustering increases proportionally to developmental progression. These spatially correlated HMRs are enriched for unique stage-relevant gene ontologies, trait-associated genetic heritability, and ChromHMM annotations, implying distinct regulatory roles compared to their unclustered counterparts.

Previous investigations into enhancers describe subsets of clustering enhancers, including super-enhancers and hub enhancers [26, 80]. Super-enhancers that have been defined by H3K27 acetylation levels or by TF binding often consist of enhancer clusters. Clustering alone does not designate SEs and only a fraction of SEs is comprised of multiple enhancers units. We wanted to understand how many B cell HMR clusters also overlap super-enhancers that have been defined by ChIP-seq approaches. Our main conclusion from this analysis is that most super-enhancers also overlap clustered HMRs, but there are many more clustered HMRs than super-enhancers. One explanation for this broader phenomenon may be the finding that HMRs are often established near existing HMRs over developmental timescales. Thus, clustered HMRs can consist of regions representing both past and present enhancer activity (perhaps long after histone modifications and TF binding are lost). The establishment and maintenance of HMRs represents a unique characteristic of DNA hypomethylation compared to other more transient chromatin states. Another important consideration is that super-enhancers are defined by strength of TF binding or histone modification, which is measured on a continuous scale, whereas methylation is measured on an absolute scale.

We note that HMR clusters show patterns of hierarchical establishment that logically follow developmental paths. However, it is unclear if HMRs that persist through cell states remain epigenetically active at later stages. Clusters may include a combination of active and decommissioned, inactive enhancers recorded in HMR patterns. Murine models of early development have highlighted contrasting dynamics between spatially and functionally related enhancers during exit from pluripotency, where some require re-methylation while others retain hypomethylation [27, 28, 38, 39, 81]. These enhancers may serve to uphold cellular states during cell fate transitions. Our lab has also observed ‘vestigial’ enhancers on shorter timescales by applying ATAC-Me-seq to a differentiation time course, simultaneously measuring chromatin accessibility and DNA methylation [20, 23]; these analyses reveal a subset of regions that undergo chromatin closing while simultaneously maintaining hypomethylation levels. This is contrary to previous models of chromatin dynamics and DNA methylation that predicted methylation gain accompanies chromatin closing. These data suggest the uncoupling of chromatin accessibility and DNA methylation dynamics in a way that leads to the persistence of HMRs in chromatin inaccessible regions.

Partitioned heritability analysis of B cell HMRs established at three distinct developmental stages revealed enrichment of traits that reflect stage-relevant biology; in general, broadly shared HMRs were enriched for heritability of broader phenotypes while B cell-specific HMRs were enriched for lymphocyte-relevant traits. Each B cell HMR subset likely suffers from power limitations, representing between 3,187,775 and 9,228,469 bp, or as low as ~ 0.1% of the genome. Despite this limitation, we observe a remarkable correspondence between heritability enrichment and stage specific HMRs. This highlights the unique ability for hypo-methylation to capture information from multiple developmental timepoints; we find highly shared, lineage-shared, and cell specific heritability enrichment all within the methylome of a differentiated cell type. The genome-wide combination of stage-specific heritability signals within clusters implies the information is not only persistent through later cell stages, but also accumulated over time. The observation that DNA hypomethylation can persist through the closing of chromatin suggests that the use of H3K27ac to identify putative enhancers precludes the observation of many HMRs, a subset of which forms hierarchical clusters that record cell developmental histories.

Our findings highlight DNA hypo-methylation as a unique epigenetic mark compared to common enhancer-associated histone marks. We highlight the unique accumulation of HMRs through developmental progression into clusters, enriched for stage-relevant SNP-based heritability. Through this process, epigenetic information can be maintained state to state. Thus, our results support that the methylome presents a historical documentation of developmental choices which could assist in the prioritization and interpretation of SNP data associated with clinical traits and diseases. These conclusions may further assist in understanding the complex role of the methylome in development and epigenetic gene regulation.

## Conclusions

Here, we characterize HMR relationships both within and between developmentally diverse cell types to understand the functional significance of complex HMR patterns. We show that levels of HMR specificity across cell-types capture time point-specific branchpoints of development. Our analysis further reveals that HMRs form clusters in proximity to active genes that are important for cell identity. This is a wide-spread phenomenon and only a very small subset of HMR clusters is explained by overlapping super-enhancer annotations. Lastly, partitioned heritability revealed the functional significance of different HMR patterns linked to specific phenotypic outcomes and indicates a quantitative relationship between HMR patterns and complex trait heritability. Altogether, our findings reveal that HMRs can predict cellular phenotypes by providing genetically distinct historical records of a cell’s journey through development, ultimately providing novel insights into how DNA *hypo*-methylation mediates genome function.

## Methods

### HMR selection/exclusion dataset

DNA HMRs were obtained through the *MethBase* DNA Methylation trackhub from the UCSC Genome Browser, which references data processed through the *MethPipe* software for processing whole genome bisulfite sequencing data [31, 32]. To achieve a high-confidence genome-wide methylation dataset, cell types were included based on a minimum coverage of 10x [82]. This resulted in the selection of: *adrenal, fetal heart, fetal spinal cord, liver, macrophage, neutrophil,* and *T cell* from the NIH Roadmap Epigenomics Consortium [7]; *H1 ESC* from Lister, et al. [4]; and *B cell, neutrophil,* and *HSPC* (listed as “HSC” on the Genome Browser) from Hodges, et al*.* [17]*.* As a primary cleaning step, to focus on non-coding HMRs, we removed promoter- and exon-overlapping HMRs (discussed below in section “Clustering annotation and percentage” and Fig. S6). To do this, we combined RefSeq exon and protein-coding gene TSSs (-2000, + 1000 bp) annotations to form an exclusion BED file. Next, we referenced this file to eliminate promoter- and exon-overlapping HMRs using the *intersect* function from the *Bedtools* package with option ‘-v’ [30]. Exclusion was defined by any basepair overlap. We required a minimum of 50 bp for an HMR to be included in our analysis.

### *CD27* multiple alignment with Hi-C

Plots were generated in reference to the hg19 genome build, showing the chromosome position: Chr 12: 6,522,500 – 6,575,000. We used a page width of 7, while HMR and methylation elements had a height of 0.3 and 1.0, respectively. The multiple alignment was constructed with the *plotgardener* R package [29], using the methylation and heatmap data represented in our HMR selection dataset. Hi-C interaction score data was visualized with the *plotHicTriangle*() function from the *plotgardener* R package [29]. Contact matrices for both samples were plotted at 10 kb resolution.

### HMR dendrogram

This analysis was performed using the per-HMR average CpG numerical matrix as composed in the methylation heatmap analysis. To perform hierarchical clustering, we used the *hclust*() function with the method “ward.D2”. Colors were added using *Adobe Illustrator*.

### Methylation heatmap

Heatmaps were generated in R with the package *pheatmap* [83]. Numerical matrices representing per-HMR DNA methylation per cell type were used as input. These were generated in bash using methylation bigWig files from the *MethBase* DNA Methylation trackhub hosted on the UCSC Genome Browser [31, 32, 82]. We used the *KentUtils* binary package to convert bigWig files to bedGraph files. bedGraph score columns were used to populate a numerical matrix representing the sample-population methylation proportion at individual HMRs in rows. The HMR consensus set used here represented all HMRs, created by concatenating HMR files from all cell types and using *Bedtools merge* to combine overlapping features. The HMRs from each cell type were filtered for a minimum length of 50 bp and against the list of RefSeq TSSs (-2000/ + 1000) and exons described above. Heatmaps were generated using R package: *pheatmap* using options: *kmeans_k* = 10, *cluster_cols* = FALSE, *cuttree_rows* = 10 [83]. We also used the option “set.seed(86)” in R for reproducibility.

### Transcription factor motif enrichment analysis

The *HOMER* perl package was used to calculate transcription factor motif enrichment [84]. A background region was used to represent the merged HMR datasets of all cell types. Natural log transformed binomial *p*-values as reported by *HOMER* were used to rank motif enrichment output. *Scaled fold enrichment* was calculated by the quotient of two *HOMER* output values: [*%target/ %background]*. Top representative TFs are displayed in Fig. 1C. All TFs shown represent the top TF by rank unless the top TFs were redundant. The second ranked TF is shown for the group, “Myeloid + HSPC,” and the third ranked TF is shown for the group, “Differentiated.” All data is represented in Fig. 3C to visualize TF enrichment differences between clustered and unclustered HMRs. Data visualization is scaled by TF to show relative cell specific enrichment. Graphing was performed in R using the *ggplot2* package [85].

### *k-*means clustering gene ontology

Gene ontology was conducted using the web-based tool: GREAT [37]. Specifically, GREAT takes BED files as input and assigns gene pairs using regulatory domains around gene TSSs (extending to the nearest gene’s central domain up to a maximum extension distance). Here, we used the default gene annotation protocol from GREAT with a maximum extension of 1 Mb. For input, we supplied BED files for each *k*-means cluster representing the HMRs in each group. Standard settings for maximum region-gene distance and gene assignment were used. Top results were downloaded from the web app using the “Shown ontology data as.tsv” selection. GREAT provided output for all k-means groups except for “Differentiated,” as this group includes > 20,000 HMRs and annotates to a large number of genes that prohibits the ability to detect gene ontology enrichment. Output files were filtered to exclude the top row before import to R. Top ranked binomial test *q*-value results are displayed as bar plots using *ggplot2* and *geom_bar()*.

### Inter-HMR distances

To measure inter-HMR distances, we employed the *Bedtools closest* function with the ‘-io -d’ options to calculate the distance from each HMR to the nearest HMR per cell type [30]. Next, we extracted the output distance column to represent our observed distribution for graphing in R. To compare this distribution to random expectation, we used a script based on the process used for shuffling in Benton M.L., 2018 which uses *Bedtools closest* to calculate distances between shuffled non-coding HMRs per cell type [86, 87]. The *Bedtools closest* function takes two input files. For this analysis, the input dataset is submitted twice. A region blacklist was used to exclude placement of HMRs during the shuffle into coding space, defined by RefSeq TSSs and exons [88]; this file was also used in the HMR annotation step. We iterated the random shuffle-closest procedure 10,000 times to create a null expectation of genomic positioning given random placement. Distances per shuffle-closest were summarized as means, yielding a distribution of average distances per shuffle. Distributions were plotted using the *ggplot2* R package. Inter-HMR distance values were filtered for those at or less than 500,000 bp to allow for better resolution of the density plot. Statistical significance between expected and observed inter-HMR distance values for each cell type were calculated using the *wilcox.test*() function in R. Statistical tests were computed on the list of inter-HMR distance values less than or equal to 500 kb.

### Clustering annotation and percentage

To assess the prevalence of clustering per cell type, we utilized the same procedures outlined in Fig. S6. Unclustered HMRs (Fig. S6A) were defined as HMRs that are not within 6 kb of any other HMR. We used *Bedtools merge* with the options ‘-c’ and ‘-d 6000’ to link BED regions and output constituent counts. We then use those that have a count of one and filter against RefSeq TSSs and exons. Because this excludes promoter and exon-proximal (within 6 kb) non-coding HMRs, we also perform a more inclusive unclustered HMR annotation by filtering HMRs by RefSeq TSSs and exons before performing a *Bedtools merge* step (‘-c’, ‘-d 6000’) to identify isolated HMRs (Fig. S6B), where *Bedtools merge* reports an input BED region that was not merged with any other HMR with a value of 1. By removing RefSeq TSS- and exon-overlapping HMRs before merging regions, we can find otherwise “unclustered” HMRs that are near a genic HMR. For Fig. 2C, we subtract the total of our working unclustered HMR definition (Fig. S6A) from this more inclusive definition to deduce the count of “TSS/exon-proximal” unclustered HMRs. To find clustered HMRs that do not cross the boundaries of TSSs and exons, we first use *Bedtools complement* to generate a BED file of regions that do not overlap the RefSeq regions. We then use *Bedtools intersect* with the ‘-c’ and ‘-F 1.0 ‘ options to find a whitelist set of regions that contain two or more (for identifying clusters with exactly 2 HMRs) or 3 or more HMRs. We use *Bedtools intersect* again with the ‘-lof’ and ‘-F 1.0’ options to produce a file where each row contains two BED coordinates: one for the whitelist region and one for the individual HMR. We then use a bash script to process this file with the purpose of linking HMR regions that are within 6 kb of each other that are within the same whitelist region (without passing a TSS or exon boundary). The output includes the linked end-to-end coordinates of clusters as well as the number of HMRs in each 6 kb-linked cluster. This can then be used to determine HMR clusters with exactly 2 (Fig. S6C) or 3 or more HMRs (Fig. S6D). To find individual clustered HMRs, we used *Bedtools intersect* with the original file as the ‘-a’ file and merged cluster datasets as ‘-b’ files. For the analyses outside of Fig. 2C, the terms “clustered” and “clusters” refers to clusters of 3 or more HMRs. For Fig. 2D, data was compiled and binned into clustered (3 +) or unclustered HMRs. Denominators were defined as the total number of clustered and unclustered HMRs so that relative quantities in each cell type are visually comparable. Plots were generated with the *ggplot2* R package.

### Sankey diagram

HMR counts for each Sankey node and flow were determined using bash and the *Bedtools* suite. Nodes represent the total quantity of clustered and unclustered HMRs per cell type. Plots were generated in R using the package *networkD3* [89]. To accurately represent the total quantity of HMRs per cell type, additional nodes were input and later processed with *Adobe Illustrator*.

### Sankey gene ontology

Gene ontology was conducted using the web-based tool: GREAT [37], as with the *k*-means clustering gene ontology analysis. Here, we again used the default gene annotation protocol from GREAT. For a background file, we used the default “Whole genome” option. Standard settings for maximum region-gene distance and gene assignment were used. Top results were downloaded from the web app using the “Shown ontology data as.tsv” selection. Output files were filtered to exclude the top row before import to R, and the preceding “#” is removed from the second row. Top results ranked by binomial *q*-value are displayed as a bar plot using *ggplot2* and *geom_bar*.

### Super-enhancer annotation

GM12878 SEs were downloaded from the *Hnisz *et al*.* in hg19 as a BED file (of coordinates for both enhancers and super-enhancers) permitting comparison with clustered and all B cell HMR datasets using *Bedtools intersect *[26]. GM12878s are a well-studied Tier 1 ENCODE cell type derived from EBV-transformed B cells. SEs were selected from the “GM12878.bed” file. To use *eulerr*, input coordinates between groups must match; to accomplish this, we concatenated the GM12878 SE, B cell clustered, and B cell unclustered files before using *Bedtools* to sort and merge the BED file. We then used *Bedtools intersect* with the ‘-u’ option and the merged BED file as the ‘-a’ file to map each input BED file to the merged regions (representing a consensus list of HMRs). These were combined in R to generate a list of three BED files. Plotting was performed using the *R* package, *eulerr*, with the option ‘shape = “ellipse”’ to maintain proportionally sized ellipses.

### ChromHMM annotation

A ChromHMM 15-state annotation file was downloaded from the UCSC Genome Browser in hg19 as assayed in the GM12878 cell line [82]. Intersections were assessed using *Bedtools intersect* with the ‘-wo’ option and B cell HMRs as the ‘-a’ file with the ChromHMM BED file as the ‘-b’ file. Using R, HMR quantities per ChromHMM group were calculated as the number of HMRs that contain at least one instance of that ChromHMM group. Denominators for calculation proportions were 5974 and 17,185 for B cell clustered and unclustered HMRs, respectively. Statistical testing was performed using the z-test of proportions in R using *prop.test()*. Graphing was performed in *R* with the package, *ggplot2*.

### ATAC-STARR-seq comparison

BED files for GM12878 ATAC-STARR-seq regulatory elements were obtained from Hansen & Hodges [58] (GSE181317). HMRs were converted to GRCh38 for comparison using *liftOver* (parameters: -bedPlus = 3). We determined the number of overlaps between the datasets with *Bedtools* *intersect* (default parameters) piped to a line count command (wc -l). The proportion of overlapping HMRs was calculated as [#overlapping/#total] and then plotted with *ggplot2* in R. We performed a two-tailed, two-sample z-test of proportions with the *prop.test()* function in R to obtain *p*-values.

### Nearest-neighbor RNA-seq analyses

To determine if clustered HMRs are more likely to associate with active genes than unclustered HMRs, we measured the proportion of HMRs near “active” genes (TPM > 0) at different distances for the two HMR groups: HMR clusters that contain cell specific HMRs and unclustered cell specific HMRs. We defined coordinates for the clustered HMR region from end-to-end including all HMR constituents. To assign the nearest HMR-gene pair, we downloaded two RNA-seq datasets acquired from the ENCODEv3 release. Here, we elected to use data for GM12878s, a lymphoblastoid cell line, to match B cells as closely as possible; and we used the ENCODE Tier 1 HepG2 dataset to as a proxy for liver. We first isolated the ENSEMBL gene ID and TPM columns from each file before averaging between replicates for each cell type using the *tidyverse* package, merge(), and *rowMeans*() in R. We then used BioMart to convert ENSEMBL IDs to HUGO gene symbols to identify high-confidence protein-coding genes [90]. To provide the highest conversion rate using BioMart, we truncated the version number from the ENSEMBL IDs. We performed the conversion using the *useMart*() function to establish search parameters with options: *biomart* = “ENSEMBL_MART_ENSEMBL,” *host* = "grch37.ensembl.org,” *path* = "/biomart/martservice,” and *dataset* = "hsapiens_gene_ensembl.” This was used in conjunction with the *getBM*() function requesting the output “attributes” of “hgnc_symbol,” “strand,” “chromosome_name,” “start_position,” and “end_position.” We then filtered the output for rows that had a non-empty “hgnc_symbol” column value. This was then merged with the dataframe described above with ENSEMBL ID and averaged TPM values. We used the strand information provided from BioMart to elect a TSS from either the “start_position” or “end_position,” based on if the “strand” was “1″ or “-1″ respectively. This file was transformed into BED format using the TSS position to create a gene file with coordinate, gene ID, and TPM information.

To find the nearest active gene to HMR clusters and unclustered HMRs, we employed a strategy to first find a large pool of surrounding genes, before filtering out pairs that cross TAD boundaries and identifying the nearest gene. To do this, we assigned the surrounding gene landscape to each HMR by using *Bedtools closest* with the ‘-d’ distance option as well as the ‘-k 100’ option to output the nearest 100 genes to each HMR. We then filtered the list of HMR-TSS pairs for TAD crossing using reference TAD BED files, for “GM12878” and “Liver,” as downloaded from the 3D Genome Browser [91]. We used *Bedtools* *intersect* with the ‘-f 1.0’ option to eliminate HMR-TSS pairs that are not fully within a TAD BED coordinate range. Using R, we filtered the resulting list to represent the nearest gene to each HMR. We then determined the quantity of HMR-gene pairs under each distance threshold (10, 25, 50, 75, 100, and 150 kb) for each HMR group, separately, by filtering the single nearest neighbor dataset by the HMR-TSS distance column and counting rows. We calculated the denominator of these proportions as the total amount of HMRs input to the analysis for each HMR group for each cell type. We used the *prop.test*() function in R to compare the HMR proportions between HMR clusters that contain cell specific HMRs and unclustered cell specific HMRs at each threshold value. Output was plotted using ggplot2, *geom*_*point*(), and *geom*_*line*().

To measure the transcriptional output differences associated with clustered or unclustered HMRs, we utilized the BED files of replicate-averaged TPM values and associated ENSEMBL IDs. We found the 2 nearest neighboring genes to each HMR using *Bedtools closest* with the ‘-d’ distance option to output HMR-TSS distances and the ‘-k 2’ option to limit output to the two nearest TSSs. We then filtered the list of HMR-TSS pairs for TAD crossing using reference TAD BED files, for “GM12878” and “Liver,” as downloaded from the 3D Genome Browser [91]. We used *Bedtools intersect* with the ‘-f 1.0’ option to eliminate HMR-TSS pairs not fully within a TAD BED coordinate range. We used R to eliminate gene redundancy within the clusters and unclustered datasets, separately. Statistical testing was performed using the *wilcox*.*test*() function in R. TPMs were plotted using *ggplot2* and *geom*_*boxplot*().

### S-LDSC

Stratified LD-score regression was performed with S-*LDSC* using the appropriate python scripts distributed by the Price lab (https://github.com/bulik/ldsc) [92]. Reference base annotation files were downloaded from the Price repository (Phase 3, version 2.2 annotations). We used the appropriate reference files coordinating with the 1000 Genomes baseline v2.2 scores and HapMap 3 SNPs (https://alkesgroup.broadinstitute.org/LDSCORE/). Summary statistics were collected from both the Price lab (https://alkesgroup.broadinstitute.org/LDSCORE/independent_sumstats/) as well as the Neale lab heritability repository (https://nealelab.github.io/UKBB_ldsc/index.html) [72]. Traits were obtained based on their determined relevance to either broad cell-agnostic etiology or to biology specifically relating to B cells or Liver. This provided us the ability to determine specificity of results associated with varying cell specificity of HMRs. In total, we assessed 79 traits as described in Table S5. The primary S-LDSC program was run per HMR annotation per trait. Results for individual traits were tabularized per HMR annotation. Results were visualized using *ggplot* in R with the functions *geom_point* and *case_when* for conditional coloring. To determine B cell developmentally derived HMRs, we used *Bedtools intersect* to compare HMR files. H1 ESC-derived B cell HMRs were defined by B cell HMR coordinates and had to had overlap with HMRs from HSPC as well as H1 ESC, together. HSPC-derived B cell HMRs had to have overlap with HSPC HMRs but not H1 ESC HMRs. B cell-specific HMRs had to have no overlap with any HMRs from the collection of adrenal gland, liver, fetal heart, fetal spinal cord, H1 ESC, HSPC, macrophage, neutrophil, and T cell HMRs. In the clustering analysis, all clustered or all unclustered HMRs were used. Liver HMRs were defined as H1 ESC-derived based on any overlap with H1 ESC HMRs. Cell specific Liver HMRs were also defined against the same comparative cell type collection used with B cell for this specific analysis. Annotations used to compare against HMR groups were selected from those included in the “baselineLF_v2.2.UKB.tar.gz” from the Price lab LD-score website. Annotations were selected for their relevance to enhancers; CTCF, a ubiquitous transcription factor, was included as a negative control for cell specific disease enrichment.

### WebGestalt gene ontology analysis

Developmentally grouped B cell HMR BED files, as used in the S-LDSC analysis, were used as input in addition to BED files for all B cell clustered or all B cell unclustered HMRs. GREAT input was used to identify nearest neighbor genes in hg19 [37]. We used the default gene assignment parameters under “Association rule settings” called “Basal plus extension,” which in most cases replicates a two-nearest neighbor gene association strategy. In the web app, we downloaded the “Gene—> genomic region association table” file from the “genomic region-gene associations” page. Gene symbols were extracted from the GREAT input downloaded files using the first column. These were input into the WebGestalt web app to perform an over-representation analysis on the disease functional database, GLAD4U [73, 74, 93]. For a reference gene set, we selected “genome protein-coding.” Results were downloaded, and the enrichment values file was used to plot enrichment ratio values for top diseases in R using *ggplot2.*

### Supplementary Information

**Additional file 1: Figure S1.** HMR lengths by cell type. Density plot of HMR lengths (in bp) by cell type. The x-axis of the plot is visually limited to the range of 0 to 5000 bp for visibility.

** Additional file 2: Figure S2.** Hierarchical clustering of HMRs by average methylation per cell type. Dendrogram of average CpG methylation across HMRs per cell type. The input matrix used for the *k*-means clustering heatmap in Fig. 1 was used for input to the R program, *ggdendro*. Distance was measured with the “euclidean” option, and hierarchical clustering was performed with the ward.D2 method.

** Additional file 3: Figure S3.** Dotplot of elbow method to determine appropriate number of *k*-means for methylation heatmap. Figure displays within sum of squares estimates for clusters at each value of *k*-means group amount from 1 to 12. Estimates are derived from the *kmeans*() function in R.

** Additional file 4: Figure S4.** Bargraph of GREAT gene ontology results by methylation heatmap *k*-means cluster. GREAT gene ontology enrichments are shown for cluster groups from the heatmap in Fig. 1B [37]. Results from the top 3 by hypergeometric FDR *q*-value are displayed. The x-axis shows the hypergeometric *q*-values. The cluster groups shown include (A) “Early developmental,” (B) “Fetal,” (C) “Liver,” (D) “Myeloid,” (E) “T cell-specific,” (F) “B cell-specific,” (G) “All,” (H) “Hematopoietic,” and (I) “Myeloid + HSPC.”

** Additional file 5: Figure S5.** HMR cluster lengths are consistent across cell types. The graph shows the lengths of HMR clusters, end-to-end, per cell type. Data is represented by both a violin plot and boxplot. The boxplot shows the interquartile range, and the bold black line shows the median value per cell type. The red dotted line shows the value 10,000 bp, which approximates the mean cluster length of 9764.59 bp, measured across the cell types: H1 ESC, fetal heart, fetal spinal cord, adrenal gland, liver, HSPC, macrophage, neutrophil, T cell, and B cell.

** Additional file 6: Figure S6.** Schematic of HMR definitions and annotation. Visual graphic of HMR definitions for groups: (A) unclustered, (B) unclustered:TSS/exon-proximal, (C) clusters of 2 HMRs, and (D) clusters of 3+ HMRs. Gene tracks are not to scale.

** Additional file 7: Figure S7.** Euler plot comparing B cell HMRs with open chromatin. Euler plot of all B cell HMRs and open chromatin defined by DNase I hypersensitivity sites in GM12878 cells. The DNase file was downloaded from the UCSC Genome Browser Table Browser using the following main settings: *clade*: “mammal”; *genome*: “human”; *assembly*: “Feb 2009 (GRCh37/hg19)”, *group*: “Regulation”, *track*: “Duke DNaseI HS”, *table*: “GM12878 Pk (wgEncodeOpenChromDnaseGm12878Pk)” [ENCODE file ID: ENCFF001UVC] [82]. The values, 13573 and 20497, represent count values for HMRs. The value 102228 represents a count for open chromatin regions.

** Additional file 8: Figure S8.** HMR proportions near active genes and boxplots comparing gene expression and distance near clustered and unclustered HMRs. (A) Point and line graph of the percentage of HMRs that are found in HMR-gene single nearest neighbor pairs at different distances. HMRs are grouped by HMR clusters that contain a cell-specific HMR and unclustered cell-specific HMRs. Denominators for the HMR clusters, unclustered (including TSS/exon-proximal), and unclustered HMR groups are 444, 2040, and 1621, respectively. *p*-values are derived from a z-test of proportions to test the fraction of HMRs represented below each threshold distance. (B) Point and line graph of the percentage of HMRs that are found in HMR-Gene single nearest neighbor pairs at different distances. HMRs are grouped by HMR clusters that contain a cell-specific HMR and unclustered cell-specific HMRs. Denominators for the HMR clusters and unclustered HMR groups are 798 and 5424, respectively. Counts below the graph represent the cumulative amount of genes below each threshold per HMR group. *p*-values are derived from a z-test of proportions to test the fraction of HMRs represented below each threshold distance. (C) Boxplot of normalized read counts of nearest neighbor RefSeq protein-coding genes to clustered and unclustered Liver HMRs. Nearest neighbor genes were filtered for TAD boundary crossing. Results for liver are also displayed in (D) for all genes, but binned by distance between the HMR and nearest gene. Statistical significance was measured by a Wilcoxon rank sum test.

** Additional file 9: Figure S9.** S-LDSC identifies Liver HMR annotation-specific trait enrichments. Volcano-style plots of S-LDSC partitioned heritability results across 79 traits are shown for two liver HMR groups: H1 ESC-derived and cell-specific. HMRs are ordered by the developmentally distinct cell type in which they were established. Each HMR group was tested for enrichment of genetic heritability with a standard set of 98 base annotations against traits that include both clinical diseases as well as clinical lab values. Negative enrichment values were clipped to the lowest positive enrichment value for each row of plots (A: 0.02781896; B: 0.03787533). The size of each point represents the -log_10_*p*-value of the enrichment, and the color shows the log_10_enrichment value. Points with a *p*-value <= 0.05 or an enrichment > 10 are labeled by their trait name where available.

** Additional file 10: Figure S10.** Disease ontology for developmentally specific and clustered B cell HMRs. Lollipop plots show top ten disease ontology enrichments as analyzed through WebGestalt with default parameters. The x-axis shows enrichment ratios, and the y-axis displays disease ontologies sourced from the GLAD4U disease database [93]. The y-axis is sorted by enrichment value. The color for each bar represents the p-value for that trait. Individual graphs show results from B cell HMR developmental and clustering groups: (A) H1 ESC-derived, (B) HSPC-derived, (C) cell-specific, (D) clustered and (E) unclustered.

** Additional file 11: Figure S11.** S-LDSC B cell by trait across genomic annotations. Point and line plots of S-LDSC enrichment values by annotation group per trait. The x-axis represents enrichment values, and the y-axis displays genomic annotations. Points show enrichment point estimates and lines display 95% confidence intervals. The red dotted line marks an enrichment score of 0. Annotation groups include popular enhancer-associated genomic annotations such as ancient human enhancer sequence age, FANTOM 5 enhancers, eQTLs, super-enhancers, and the H3K27ac histone mark [72]. Genomic controls were also included, such as phastCons 46-way annotations as well as promoters and CTCF sites. These graphs include data from (A) developmentally derived B cell HMRs. (B) This graph shows S-LDSC results for alanine transaminase. The data includes the annotations from (A) in addition to developmentally derived Liver HMRs.

** Additional file 12: Table S1.** Table of coverage values for WGBS datasets per cell type.

** Additional file 13: Table S2.** Number of HMRs after preliminary filters.

** Additional file 14: Table S3.** Inter-HMR lengths by cell type.

** Additional file 15: S4 Table.** Clustering group region counts by clustering distance (bp).

** Additional file 16: Table S5.** List of 79 summary statistic files used for S-LDSC analyses.

** Additional file 17: Table S6.** Program and package versions.

## Data Availability

Data analysis tools are publicly available. Versions of software and packages are summarized in Table S6. The analysis code involved in this paper has been made publicly available at https://github.com/HodgesGenomicsLab/HMRPatterns. The primary HMR, methylation, and bisulfite-sequencing read datasets can be found processed and distributed from the MethBase methylation database [31, 32]. The accession number for the raw human GM12878 SE H3K27ac ChIP-seq data is GSM733771. The accession number for the H9 ESC Hi-C data is GSM3262957. The raw and processed sequencing data for ATAC-STARR-seq calls is available under accession number GSE181317. ENCODEv3 RNA-seq gene quantifications for GM12878 rep 1 and 2 are available through accession numbers ENCFF873VWU and ENCFF345SHY, respectively. ENCODEv3 RNA-seq gene quantifications for HepG2 rep 1 and 2 are available through accession numbers ENCFF974MUO and ENCFF649AHO, respectively. The GM12878 Hi-C data was found through the 3D Genome Browser and originates from the publication Rao et al., 2014 (GEO: GSE36525) [62]. The H9 ESC Hi-C data was found through the 4D Nucleome Data Portal and represents data produced by Zhang et al., 2019 (GEO: GSM3262957) [63, 94].

## Abbreviations

HMR: Hypomethylated region
TF: Transcription factor
TSS: Transcription start site
SE: Super-enhancer
LD: Linkage disequilibrium
S-LDSC: Stratified linkage disequilibrium score regression
GWAS: Genome-wide association study
SNP: Single nucleotide polymorphism
ATAC-STARR-seq: Assay for transposase-accessible chromatin with self-transcribing active regulatory region sequencing
DNAme: DNA methylation
WGBS: Whole genome bisulfite sequencing
HSPC: Hematopoietic stem and progenitor cells
ESC: Embryonic stem cells
