# Supplementary material for: Cross-tissue patterns of DNA hypomethylation reveal genetically distinct histories of cell development
Source: BMC Genomics. 2023 Oct 19;24:623. doi: 10.1186/s12864-023-09622-9 (PMC10588161; doi:10.1186/s12864-023-09622-9)
Supplement: Supplementary file 1 — Additional file 1: Figure S1. HMR lengths by cell type. Density plot of HMR lengths (in bp) by cell type. The x-axis of the plot is visually limited to the range of 0 to 5000 bp for visibility. [file 12864_2023_9622_MOESM1_ESM.pdf]

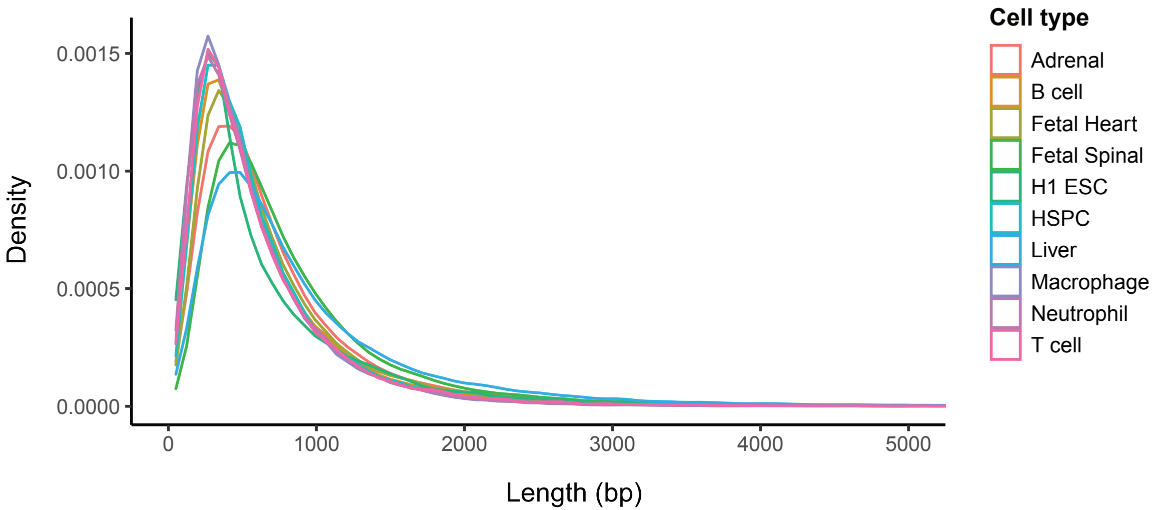

Figure S1. HMR lengths by cell type.
Density plot of HMR lengths (in bp) by cell type. The x-axis of the plot is visually limited to the range of 0 to 5000 bp for visibility.
