# Supplementary material for: Cross-tissue patterns of DNA hypomethylation reveal genetically distinct histories of cell development
Source: BMC Genomics. 2023 Oct 19;24:623. doi: 10.1186/s12864-023-09622-9 (PMC10588161; doi:10.1186/s12864-023-09622-9)
Supplement: Supplementary file 2 — Additional file 2: Figure S2. Hierarchical clustering of HMRs by average methylation per cell type. Dendrogram of average CpG methylation across HMRs per cell type. The input matrix used for the k-means clustering heatmap in Fig. 1 was used for input to the R program, ggdendro. Distance was measured with the “euclidean” option, and hierarchical clustering was performed with the ward.D2 method. [file 12864_2023_9622_MOESM2_ESM.pdf]

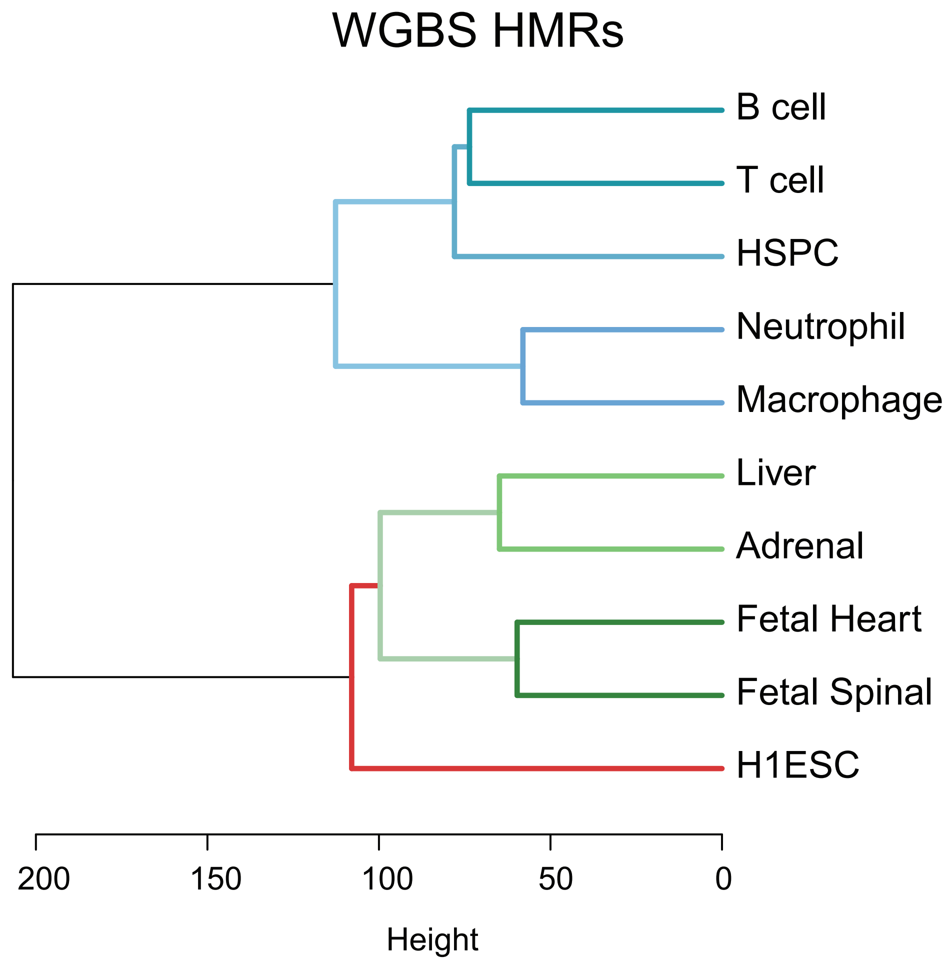

Figure S2. Hierarchical clustering of HMRs by average methylation per cell type. Dendrogram of average CpG methylation across HMRs per cell type. The input matrix used for the *k*-means clustering heatmap in Fig. 1 was used for input to the R program, ggdendro. Distance was measured with the “euclidean” option, and hierarchical clustering was performed with the ward.D2 method.
