# Supplementary material for: Cross-tissue patterns of DNA hypomethylation reveal genetically distinct histories of cell development
Source: BMC Genomics. 2023 Oct 19;24:623. doi: 10.1186/s12864-023-09622-9 (PMC10588161; doi:10.1186/s12864-023-09622-9)
Supplement: Supplementary file 3 — Additional file 3: Figure S3. Dotplot of elbow method to determine appropriate number of k-means for methylation heatmap. Figure displays within sum of squares estimates for clusters at each value of k-means group amount from 1 to 12. Estimates are derived from the kmeans() function in R. [file 12864_2023_9622_MOESM3_ESM.pdf]

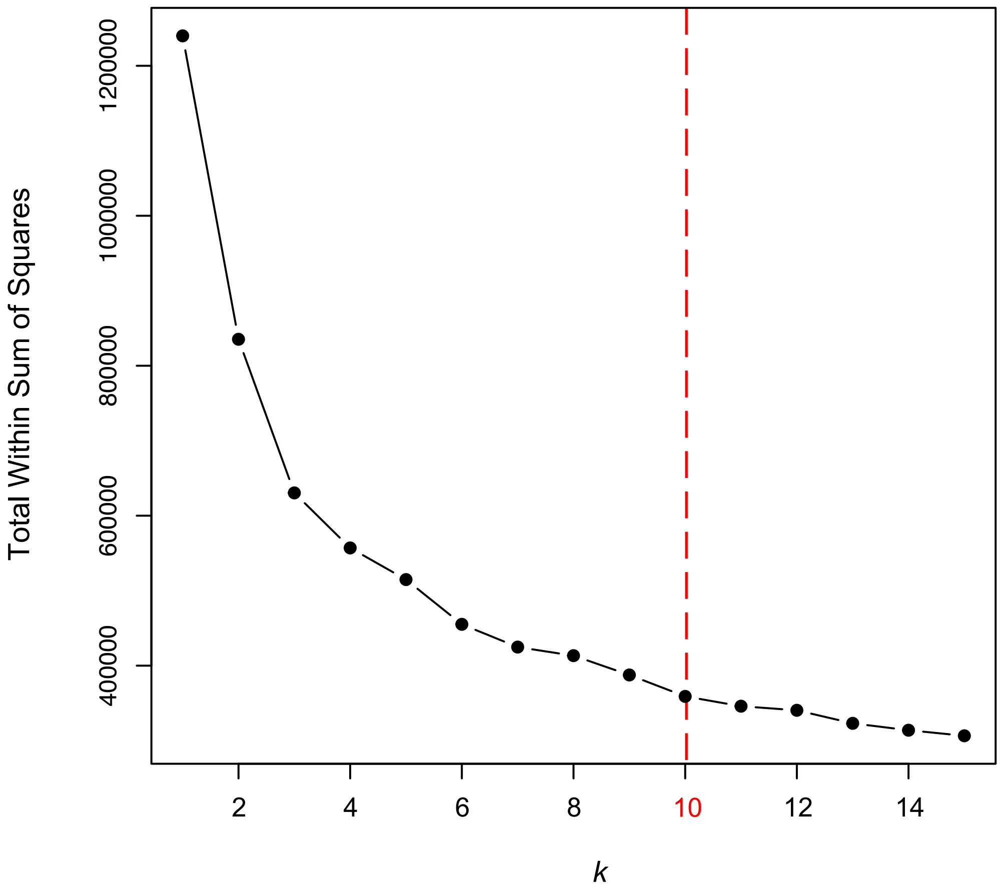

Figure S3. Dotplot of elbow method to determine appropriate number of *k*-means for methylation heatmap.
Figure displays within sum of squares estimates for clusters at each value of *k*-means group amount from 1 to 12. Estimates are derived from the kmeans() function in R.
