# Supplementary material for: Cross-tissue patterns of DNA hypomethylation reveal genetically distinct histories of cell development
Source: BMC Genomics. 2023 Oct 19;24:623. doi: 10.1186/s12864-023-09622-9 (PMC10588161; doi:10.1186/s12864-023-09622-9)
Supplement: Supplementary file 4 — Additional file 4: Figure S4. Bargraph of GREAT gene ontology results by methylation heatmap k-means cluster. GREAT gene ontology enrichments are shown for cluster groups from the heatmap in Fig. 1B [37]. Results from the top 3 by hypergeometric FDR q-value are displayed. The x-axis shows the hypergeometric q-values. The cluster groups shown include (A) “Early developmental,” (B) “Fetal,” (C) “Liver,” (D) “Myeloid,” (E) “T cell-specific,” (F) “B cell-specific,” (G) “All,” (H) “Hematopoietic,” and (I) “Myeloid + HSPC.” [file 12864_2023_9622_MOESM4_ESM.pdf]

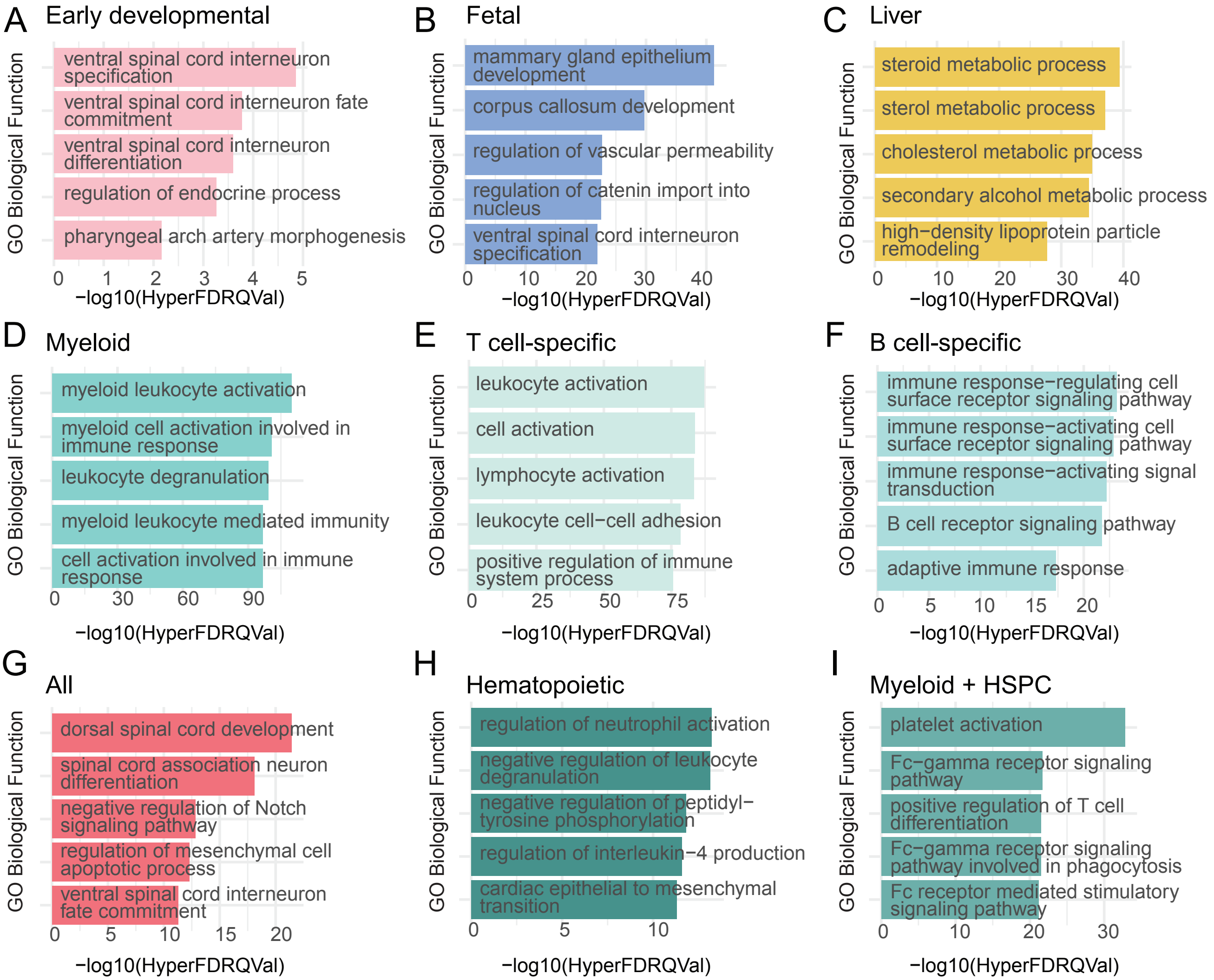

Figure S4. Bargraph of GREAT gene ontology results by methylation heatmap *k*-means cluster. GREAT gene ontology enrichments are shown for cluster groups from the heatmap in Fig. 1B (34). Results from the top 3 by hypergeometric FDR *q*-value are displayed. The x-axis shows the hypergeometric *q*-values. The cluster groups shown include (A) "Early developmental," (B) "Fetal," (C) "Liver," (D) "Myeloid," (E) "T cell-specific," (F) "B cell-specific," (G) "All," (H) "Hematopoietic," and (I) "Myeloid + HSPC."
