# Supplementary material for: Cross-tissue patterns of DNA hypomethylation reveal genetically distinct histories of cell development
Source: BMC Genomics. 2023 Oct 19;24:623. doi: 10.1186/s12864-023-09622-9 (PMC10588161; doi:10.1186/s12864-023-09622-9)
Supplement: Supplementary file 5 — Additional file 5: Figure S5. HMR cluster lengths are consistent across cell types. The graph shows the lengths of HMR clusters, end-to-end, per cell type. Data is represented by both a violin plot and boxplot. The boxplot shows the interquartile range, and the bold black line shows the median value per cell type. The red dotted line shows the value 10,000 bp, which approximates the mean cluster length of 9764.59 bp, measured across the cell types: H1 ESC, fetal heart, fetal spinal cord, adrenal gland, liver, HSPC, macrophage, neutrophil, T cell, and B cell. [file 12864_2023_9622_MOESM5_ESM.pdf]

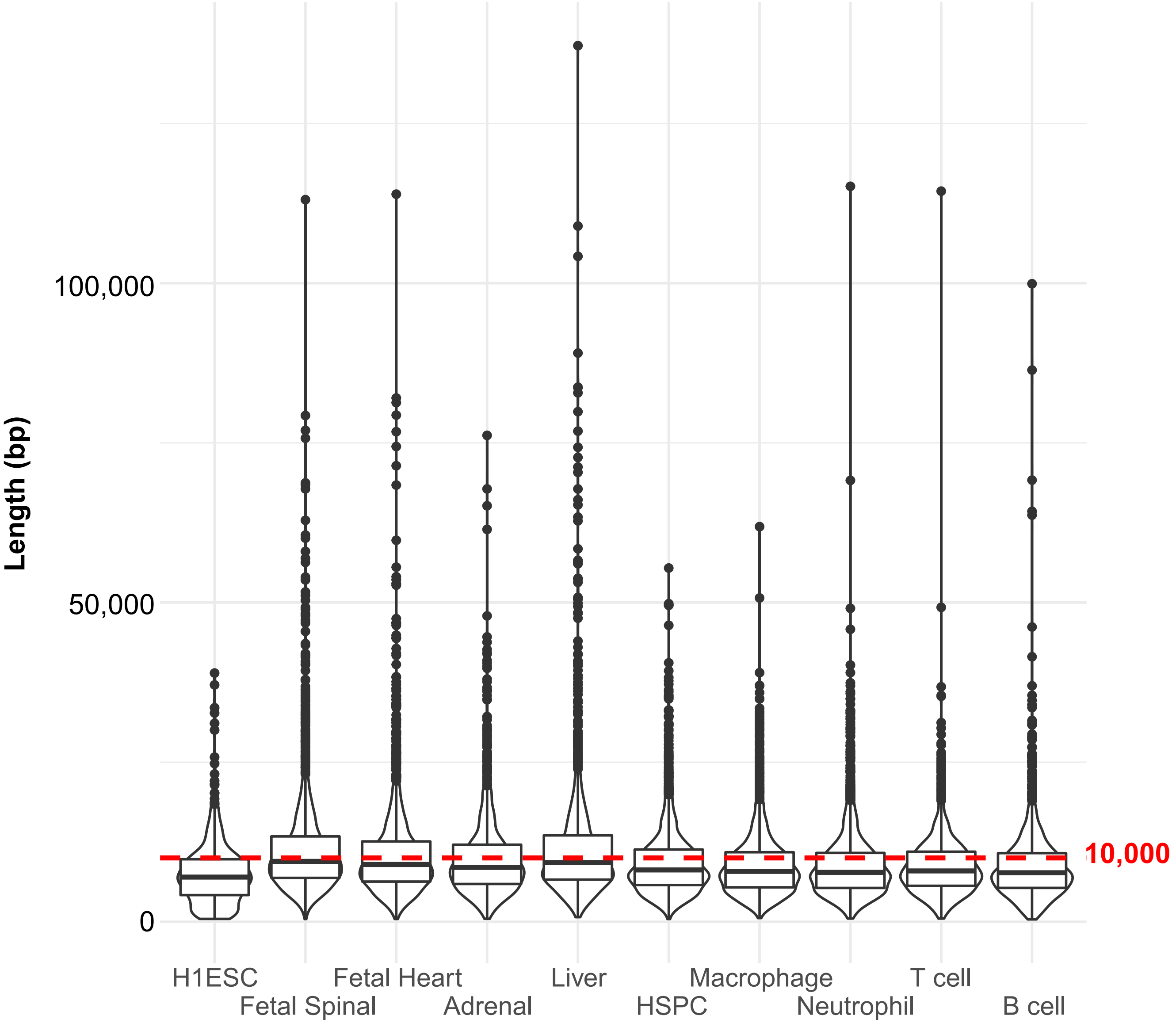

Figure S5. HMR cluster lengths are consistent across cell types.
The graph shows the lengths of HMR clusters, end-to-end, per cell type. Data is represented by both a violin plot and boxplot. The boxplot shows the interquartile range, and the bold black line shows the median value per cell type. The red dotted line shows the value 10,000 bp, which approximates the mean cluster length of 9764.59 bp, measured across the cell types: H1 ESC, fetal heart, fetal spinal cord, adrenal gland, liver, HSPC, macrophage, neutrophil, T cell, and B cell.
