# Supplementary material for: Cross-tissue patterns of DNA hypomethylation reveal genetically distinct histories of cell development
Source: BMC Genomics. 2023 Oct 19;24:623. doi: 10.1186/s12864-023-09622-9 (PMC10588161; doi:10.1186/s12864-023-09622-9)
Supplement: Supplementary file 6 — Additional file 6: Figure S6. Schematic of HMR definitions and annotation. Visual graphic of HMR definitions for groups: (A) unclustered, (B) unclustered:TSS/exon-proximal, (C) clusters of 2 HMRs, and (D) clusters of 3+ HMRs. Gene tracks are not to scale. [file 12864_2023_9622_MOESM6_ESM.pdf]

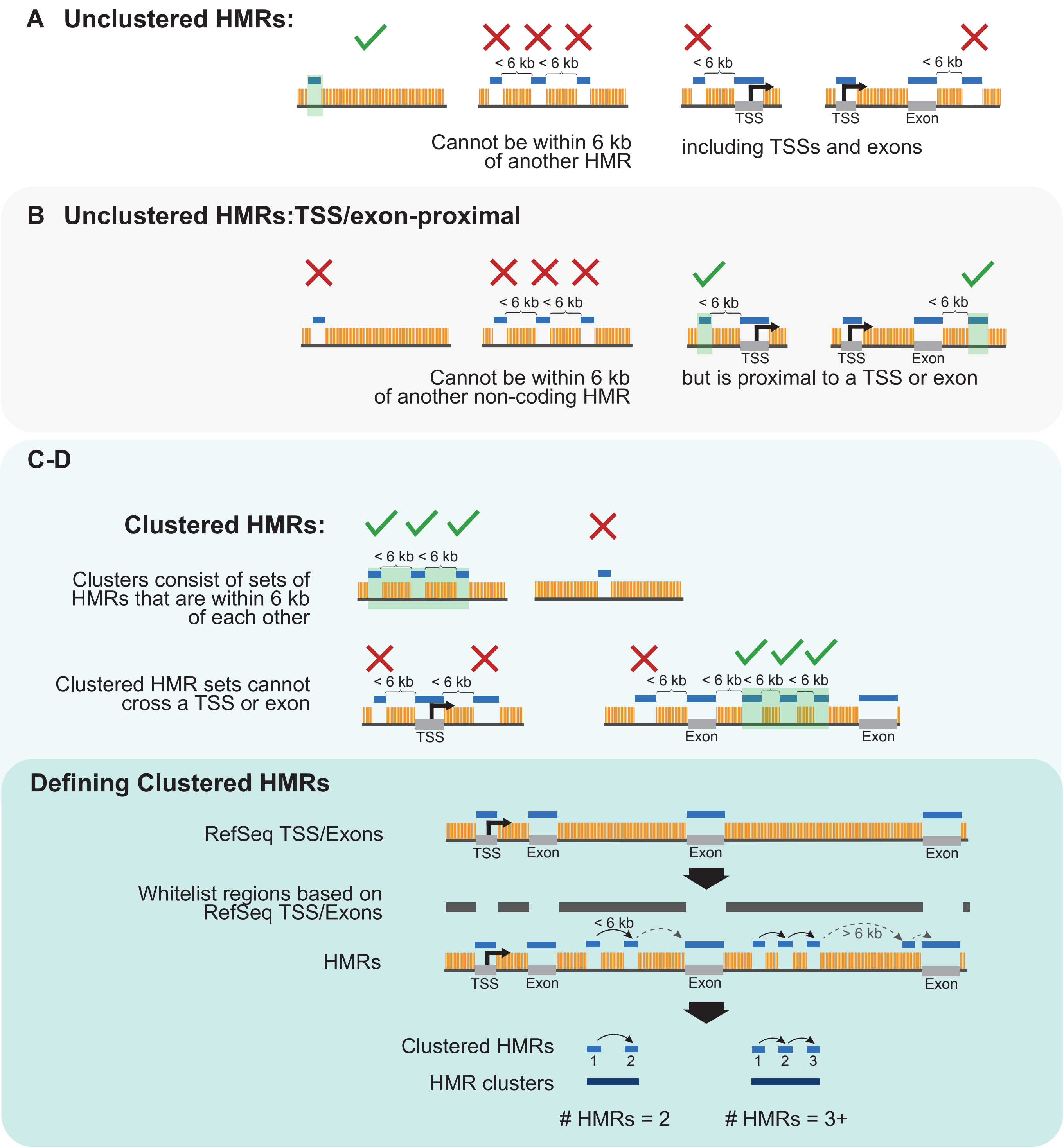

Figure S6. Schematic of HMR definitions and annotation.
Visual graphic of HMR definitions for groups: (A) unclustered, (B) unclustered:TSS/exon-proximal, (C) clusters of 2 HMRs, and (D) clusters of 3+ HMRs. Gene tracks are not to scale.
