# Supplementary material for: Cross-tissue patterns of DNA hypomethylation reveal genetically distinct histories of cell development
Source: BMC Genomics. 2023 Oct 19;24:623. doi: 10.1186/s12864-023-09622-9 (PMC10588161; doi:10.1186/s12864-023-09622-9)
Supplement: Supplementary file 7 — Additional file 7: Figure S7. Euler plot comparing B cell HMRs with open chromatin. Euler plot of all B cell HMRs and open chromatin defined by DNase I hypersensitivity sites in GM12878 cells. The DNase file was downloaded from the UCSC Genome Browser Table Browser using the following main settings: clade: “mammal”; genome: “human”; assembly: “Feb 2009 (GRCh37/hg19)”, group: “Regulation”, track: “Duke DNaseI HS”, table: “GM12878 Pk (wgEncodeOpenChromDnaseGm12878Pk)” [ENCODE file ID: ENCFF001UVC] [82]. The values, 13573 and 20497, represent count values for HMRs. The value 102228 represents a count for open chromatin regions. [file 12864_2023_9622_MOESM7_ESM.pdf]

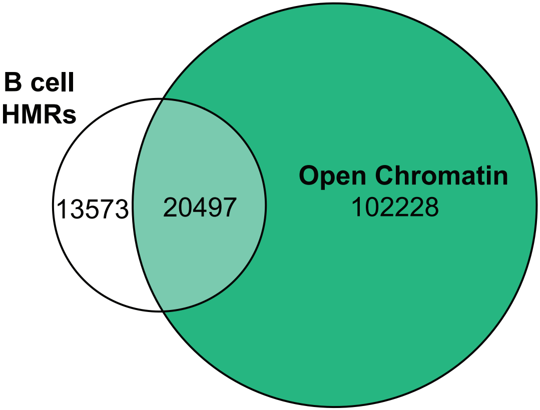

Figure S7. Euler plot comparing B cell HMRs with open chromatin.
Euler plot of all B cell HMRs and open chromatin defined by DNase I hypersensitivity sites in GM12878 cells. The DNase file was downloaded from the UCSC Genome Browser Table Browser using the following main settings: clade: “mammal”; genome: “human”; assembly: “Feb 2009 (GRCh37/hg19)”, group: “Regulation”, track: “Duke DNaseI HS”, table: “GM12878 Pk (wgEncodeOpenChromDnaseGm12878Pk)” [ENCODE file ID: ENCFF001UVC] (80). The values, 13573 and 20497, represent count values for HMRs. The value102228 represents a count for open chromatin regions.
