# Supplementary material for: Cross-tissue patterns of DNA hypomethylation reveal genetically distinct histories of cell development
Source: BMC Genomics. 2023 Oct 19;24:623. doi: 10.1186/s12864-023-09622-9 (PMC10588161; doi:10.1186/s12864-023-09622-9)
Supplement: Supplementary file 8 — Additional file 8: Figure S8. HMR proportions near active genes and boxplots comparing gene expression and distance near clustered and unclustered HMRs. (A) Point and line graph of the percentage of HMRs that are found in HMR-gene single nearest neighbor pairs at different distances. HMRs are grouped by HMR clusters that contain a cell-specific HMR and unclustered cell-specific HMRs. Denominators for the HMR clusters, unclustered (including TSS/exon-proximal), and unclustered HMR groups are 444, 2040, and 1621, respectively. p-values are derived from a z-test of proportions to test the fraction of HMRs represented below each threshold distance. (B) Point and line graph of the percentage of HMRs that are found in HMR-Gene single nearest neighbor pairs at different distances. HMRs are grouped by HMR clusters that contain a cell-specific HMR and unclustered cell-specific HMRs. Denominators for the HMR clusters and unclustered HMR groups are 798 and 5424, respectively. Counts below the graph represent the cumulative amount of genes below each threshold per HMR group. p-values are derived from a z-test of proportions to test the fraction of HMRs represented below each threshold distance. (C) Boxplot of normalized read counts of nearest neighbor RefSeq protein-coding genes to clustered and unclustered Liver HMRs. Nearest neighbor genes were filtered for TAD boundary crossing. Results for liver are also displayed in (D) for all genes, but binned by distance between the HMR and nearest gene. Statistical significance was measured by a Wilcoxon rank sum test. [file 12864_2023_9622_MOESM8_ESM.pdf]

Scott et al. Supplementary Figure 8

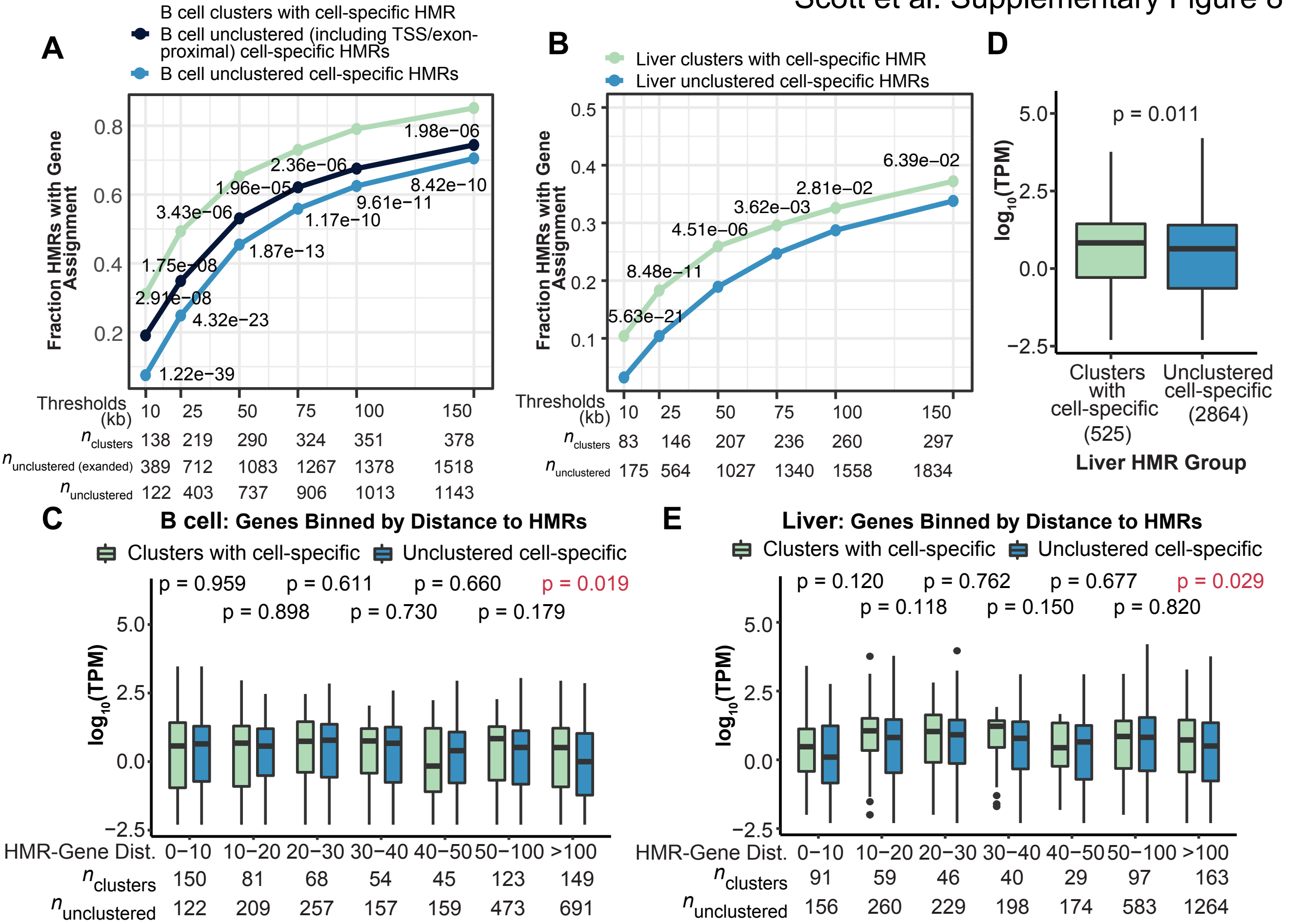

Figure S8. HMR proportions near active genes and boxplots comparing gene expression and distance near clustered and unclustered HMRs.
A) Point and line graph of the percentage of HMRs that are found in HMR-Gene single nearest neighbor pairs at different distances. HMRs are grouped by HMR clusters that contain a cell-specific HMR and unclustered cell-specific HMRs. Denominators for the HMR clusters, unclustered (including TSS/exon-proximal), and unclustered HMR groups are 444, 2040, and 1621, respectively. *p*-values are derived from a z-test of proportions to test the fraction of HMRs represented below each threshold distance. (B) Point and line graph of the percentage of HMRs that are found in HMR-Gene single nearest neighbor pairs at different distances. HMRs are grouped by HMR clusters that contain a cell-specific HMR and unclustered cell-specific HMRs. Denominators for the HMR clusters and unclustered HMR groups are 798 and 5424, respectively. Counts below the graph represent the cumulative amount of genes below each threshold per HMR group. *p*-values are derived from a z-test of proportions to test the fraction of HMRs represented below each threshold distance. (C) Boxplot of normalized read counts of nearest neighbor RefSeq protein-coding genes to clustered and unclustered Liver HMRs. Nearest neighbor genes were filtered for TAD boundary crossing. Results for liver are also displayed in (D) for all genes, but binned by distance between the HMR and nearest gene. Statistical significance was measured by a Wilcoxon rank sum test.
