# Supplementary material for: Cross-tissue patterns of DNA hypomethylation reveal genetically distinct histories of cell development
Source: BMC Genomics. 2023 Oct 19;24:623. doi: 10.1186/s12864-023-09622-9 (PMC10588161; doi:10.1186/s12864-023-09622-9)
Supplement: Supplementary file 9 — Additional file 9: Figure S9. S-LDSC identifies Liver HMR annotation-specific trait enrichments. Volcano-style plots of S-LDSC partitioned heritability results across 79 traits are shown for two liver HMR groups: H1 ESC-derived and cell-specific. HMRs are ordered by the developmentally distinct cell type in which they were established. Each HMR group was tested for enrichment of genetic heritability with a standard set of 98 base annotations against traits that include both clinical diseases as well as clinical lab values. Negative enrichment values were clipped to the lowest positive enrichment value for each row of plots (A: 0.02781896; B: 0.03787533). The size of each point represents the -log10p-value of the enrichment, and the color shows the log10enrichment value. Points with a p-value <= 0.05 or an enrichment > 10 are labeled by their trait name where available. [file 12864_2023_9622_MOESM9_ESM.pdf]

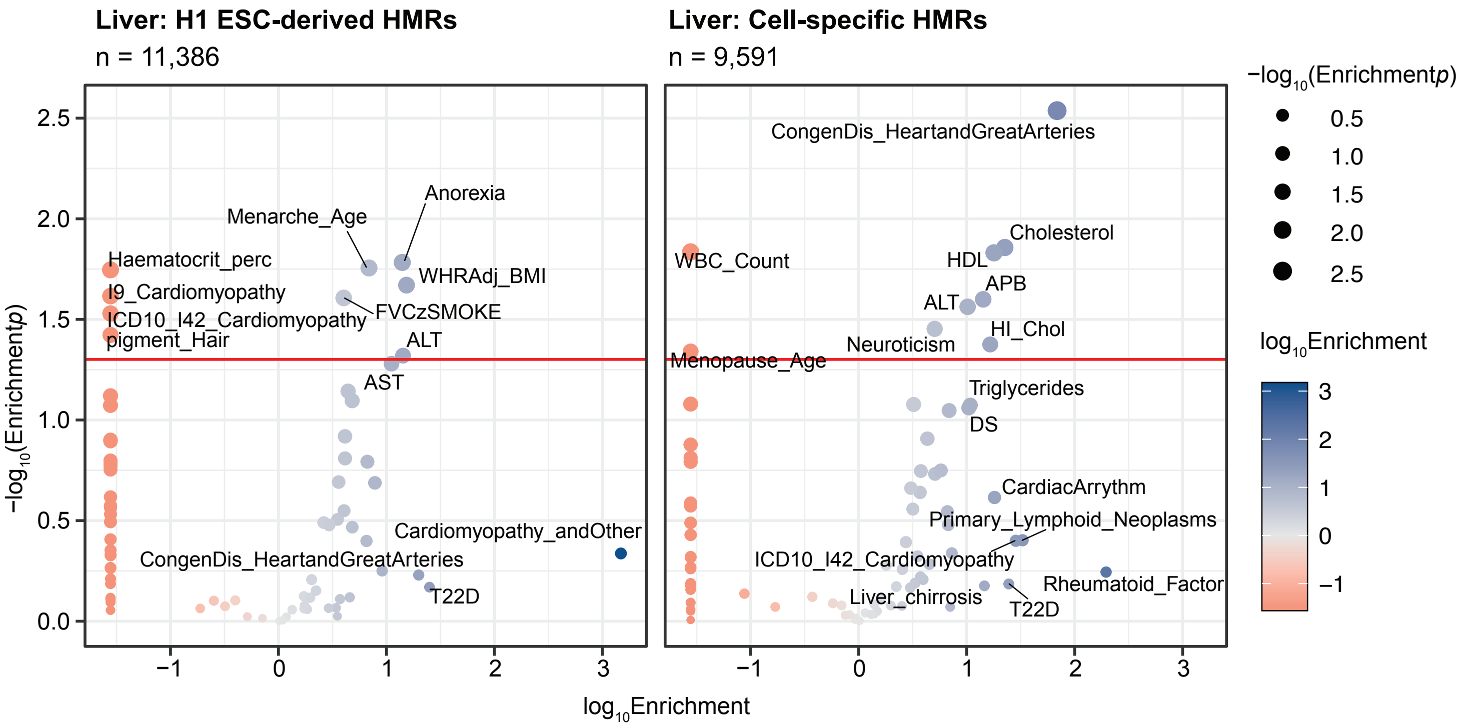

Figure S9. S-LDSC identifies Liver HMR annotation-specific trait enrichments.
Volcano-style plots of S-LDSC partitioned heritability results across 79 traits are shown for two liver HMR groups: H1 ESC-derived and cell-specific. HMRs are ordered by the developmentally distinct cell type in which they were established. Each HMR group was tested for enrichment of genetic heritability with a standard set of 98 base annotations against traits that include both clinical diseases as well as clinical lab values. Negative enrichment values were clipped to the lowest positive enrichment value for each row of plots (A: 0.02781896; B: 0.03787533). The size of each point represents the -$\log_{10}$*p*-value of the enrichment, and the color shows the $\log_{10}$enrichment value. Points with a *p*-value <= 0.05 or an enrichment > 10 are labeled by their trait name where available.
