# Supplementary material for: Cross-tissue patterns of DNA hypomethylation reveal genetically distinct histories of cell development
Source: BMC Genomics. 2023 Oct 19;24:623. doi: 10.1186/s12864-023-09622-9 (PMC10588161; doi:10.1186/s12864-023-09622-9)
Supplement: Supplementary file 10 — Additional file 10: Figure S10. Disease ontology for developmentally specific and clustered B cell HMRs. Lollipop plots show top ten disease ontology enrichments as analyzed through WebGestalt with default parameters. The x-axis shows enrichment ratios, and the y-axis displays disease ontologies sourced from the GLAD4U disease database [93]. The y-axis is sorted by enrichment value. The color for each bar represents the p-value for that trait. Individual graphs show results from B cell HMR developmental and clustering groups: (A) H1 ESC-derived, (B) HSPC-derived, (C) cell-specific, (D) clustered and (E) unclustered. [file 12864_2023_9622_MOESM10_ESM.pdf]

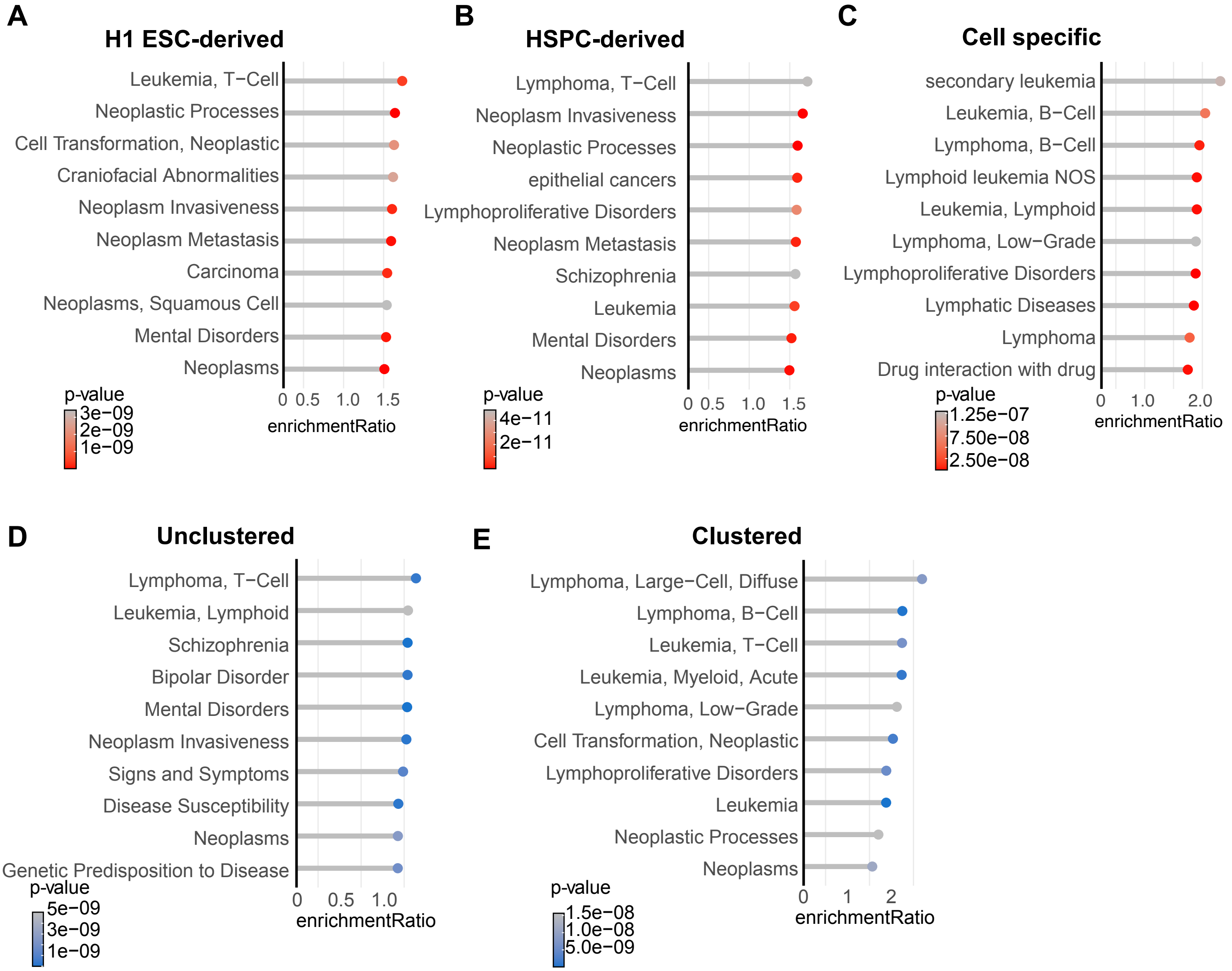

Figure S10. Disease ontology for developmentally specific and clustered B cell HMRs.
Lollipop plots show top ten disease ontology enrichments as analyzed through WebGestalt with default parameters. The x-axis shows enrichment ratios, and the y-axis displays disease ontologies sourced from the GLAD4U disease database (93). The y-axis is sorted by enrichment value. The color for each bar represents the p-value for that trait. Individual graphs show results from B cell HMR developmental and clustering groups: (A) H1 ESC-derived, (B) HSPC-derived, (C) cell-specific, (D) clustered and (E) unclustered.
