# Supplementary material for: Cross-tissue patterns of DNA hypomethylation reveal genetically distinct histories of cell development
Source: BMC Genomics. 2023 Oct 19;24:623. doi: 10.1186/s12864-023-09622-9 (PMC10588161; doi:10.1186/s12864-023-09622-9)
Supplement: Supplementary file 11 — Additional file 11: Figure S11. S-LDSC B cell by trait across genomic annotations. Point and line plots of S-LDSC enrichment values by annotation group per trait. The x-axis represents enrichment values, and the y-axis displays genomic annotations. Points show enrichment point estimates and lines display 95% confidence intervals. The red dotted line marks an enrichment score of 0. Annotation groups include popular enhancer-associated genomic annotations such as ancient human enhancer sequence age, FANTOM 5 enhancers, eQTLs, super-enhancers, and the H3K27ac histone mark [72]. Genomic controls were also included, such as phastCons 46-way annotations as well as promoters and CTCF sites. These graphs include data from (A) developmentally derived B cell HMRs. (B) This graph shows S-LDSC results for alanine transaminase. The data includes the annotations from (A) in addition to developmentally derived Liver HMRs. [file 12864_2023_9622_MOESM11_ESM.pdf]

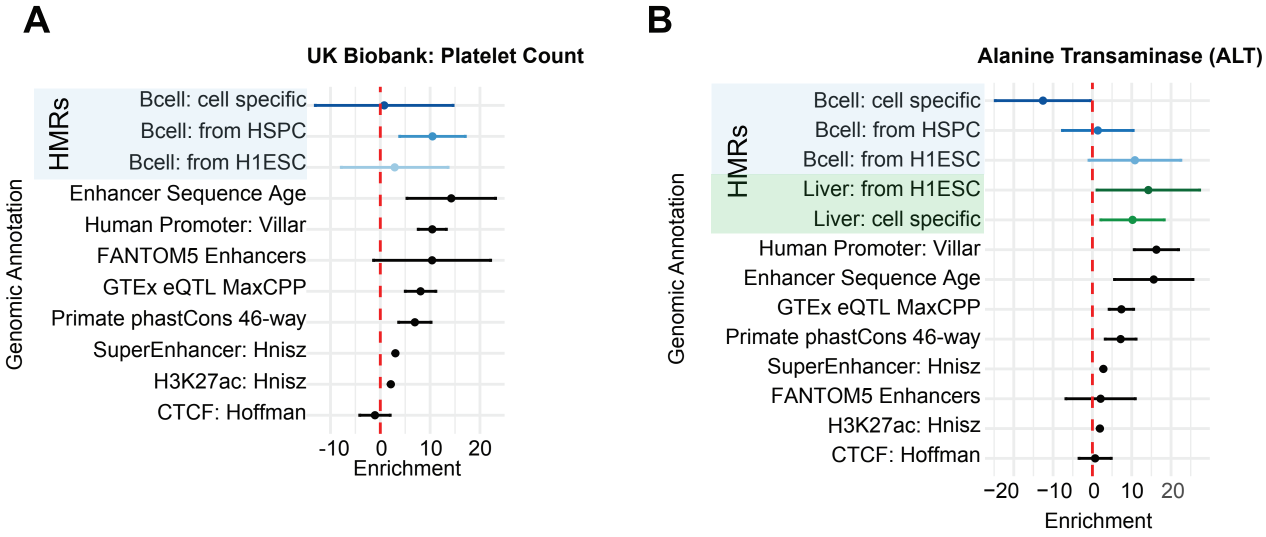


**Figure S11. S-LDSC B cell by trait across genomic annotations.**
Point and line plots of S-LDSC enrichment values by annotation group per trait . The x-axis represents enrichment values, and the y-axis displays genomic annotations. Points show enrichment point estimates and lines display 95% confidence intervals. The red dotted line marks an enrichment score of 0. Annotation groups include popular enhancer-associated genomic annotations such as ancient human enhancer sequence age, FANTOM 5 enhancers, eQTLs, super enhancers, and the H3K27ac histone mark (67). Genomic controls were also included, such as phastCons 46-way annotations as well as promoters and CTCF sites. These graphs include data from (A) developmentally derived B cell HMRs. (B) This graph shows S-LDSC results for alanine transaminase. The data includes the annotations from (A) in addition to developmentally derived Liver HMRs.
